# Green Synthesis of Silver Nanoparticles: Optimizing Green Tea Leaf Extraction for Enhanced Physicochemical Properties

**DOI:** 10.1021/acsomega.3c03775

**Published:** 2023-08-10

**Authors:** Anna Wirwis, Zygmunt Sadowski

**Affiliations:** Department of Process Engineering and Technology of Polymer and Carbon Materials, Wroclaw University of Science and Technology, Wybrzeze Wyspianskiego 27, 50-370 Wrocław, Poland

## Abstract

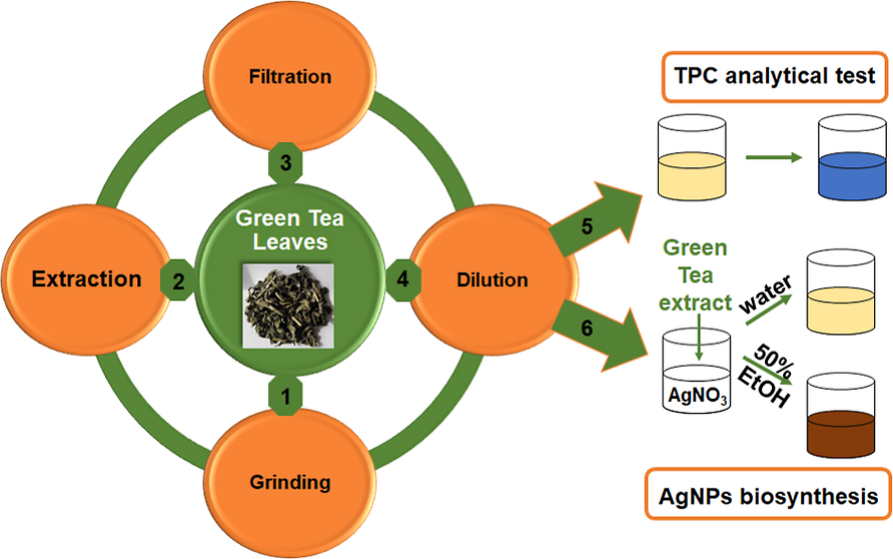

In this paper, we present the optimization of green tea leaf (*Camellia sinensis* L.) extraction, carried out using water and hydroalcoholic solvents, for the subsequent synthesis of silver nanoparticles (AgNPs). The value ranges for independent variables, including pH, time, and temperature, were selected based on single-factor experiments and used for extraction in the order presented by the Box–Behnken design. Three-dimensional response surface graphs were used to visually present the optimization results and determine the optimal extraction conditions: pH = 7, 30 min, 80 °C for water and pH = 5.5, 50 min, and 80 °C for water–ethanol. Our findings indicate that the water–ethanol mixture extracted more polyphenols. We compared the physicochemical properties of AgNPs obtained using both types of extractants via DLS and TEM analysis. We proposed a predicted mechanism for the reduction and stabilization of AgNPs based on the Fourier transform infrared data. The hydroethanolic extract leads to significant nanoparticle aggregation, which can be explained by the nucleation theory and agglomeration of nanoparticles in the presence of excess macromolecular organic substances (flocculation).

## 1 Introduction

Throughout history, numerous biologically active compounds have been identified and extracted from various plant parts that are used as valuable medicines, yet many more await discovery.^1–4^ Medicinal plants and their plant-derived bioactive substances have been reported to have anti-inflammatory, antioxidant, antibacterial, and anticancer effects, among others.^5–7^ These properties enable the treatment or alleviation of certain health conditions without the resort of potentially toxic synthetic compounds. Among this vast array of bioactive substances, polyphenols stand out as potent natural antioxidants.^8,9^ Green tea leaves (*Camellia sinensis* L.) are a popular plant source of polyphenolic compounds.^10^ The place, cultivation method, climatic conditions, and harvesting time significantly influence the chemical composition. Green tea leaves contain various bioactive substances, such as amino acids, proteins, carbohydrates, minerals, and pigments. However, the primary chemical component comprising up to 30% of the mass of dry leaves are polyphenols, particularly four catechins: epicatechin, epicatechin gallate, epigallocatechin, and epigallocatechin gallate.^11–14^ A significant advantage of extracting polyphenols from plant material is the absence of the need for a sophisticated method to aid in facilitating the extraction process. Extracting polyphenolic compounds from plant leaves is an example of a solid–liquid separation. Optimizing its crucial parameters has become necessary to transfer this process to a pilot scale. Mathematical and statistical models, including surface response techniques, are commonly employed to determine optimal conditions for obtaining extraction products efficiently, quickly, and economically. Several parameters are usually considered, such as the solid–liquid ratio, temperature, process duration, pH, and extractant type. In cases where ultrasound-assisted extraction is utilized, optimization also includes verifying the duration and power of ultrasound.^15–17^ Various leaves of plants have been extracted, and the process parameters have been optimized. Examples of these plants include *Hibiscus cannabinus* L,^15^*Lobelia rucotianifolia*,^17^*Azadirachta indica* (neem),^18–20^*Eucalyptus globulus*,^21^*Murraya koenigii* (Linn),^22^*Myrtus communis* L,^23^*Rhamnus alaternus*,^24^*Stevia redaudiana*^25^ and pure heart plant, and olive leaves.^26,27^ Green tea, known for its easy commercial availability and richness in polyphenols, is the most widely used type of tea for extraction.^12^ The procedures employing microwaves (MAE),^28–30^ ultrasound (UAE)^31^ and other green conditions^32^ are popular in the extraction process of these plant leaves beyond conventional methods. The literature has examples of extracting polyphenols from green tea leaves with the assumption of using statistical models.^14,29,31,33–37^ So far, Box–Behnken design (BBD),^35,36^ central composite design (CCD),^14,31,37^ and orthogonal assay design^29^ have been used. Additionally, the prepared extracts are tested to evaluate antioxidant activity. It is thought that the extractant and grain size of the green tea matrix often have a significant impact on the efficiency of the extraction process.^38^ Menezes and Bindes proved that a specific grain size (0.15–0.74 mm) optimized based on the statistical Turkey test is important for maximizing the extraction efficiency of polyphenols using water as an extractant.^33^ Kim and his group examined the influence of three important parameters affecting the extraction of polyphenols, including ethanol concentration (0–100%), temperature (10–70 °C), and time (3–15 min) as input values for the BBD model combination matrix.^35^ The highest antioxidant activity above 85% was observed for the highest values of time and temperature and also for an ethanol concentration of 57.7%. On the other hand, the model was applied to extraction in water, choosing biomass/solvent ratio (1:10–1:50), temperature (40–50 °C), and extraction duration (15–45 min) as independent variables.^36^ Based on CCD, Lee et al. optimized a conventional green tea extraction method using temperature, time, and ethanol concentration as initial parameters.^14^ They compared the yield and oxidative activity obtained under optimal conditions with those measured for ultrasound-assisted extraction. Thus, they proved that ultrasonication promotes the reduction of the extraction time and ethanol concentration without affecting the antioxidant activity of polyphenols. More recently, Al-Hatim and coauthors performed green tea extraction under microwave conditions using an orthogonal assay design.^29^ Statistical analysis included the following extraction conditions: microwave intensity, extraction time, biomass/water ratio, and also frequency of microwave exposure. Interestingly, the time required for extraction under the indicated conditions was only 3 min. Less common tea leaves, such as black^39^ and oolong,^40^ were also used in the extraction processes.

Both black and green tea extracts have been previously proven effective for silver nanoparticle synthesis.^41–43^ The qualitative and quantitative composition of the tea extract is the primary factor affecting the synthesis of silver nanoparticles (AgNPs). Therefore, tea fermentation processes have been optimized,^44^ and microwaves have been used during extraction.^45^ Tea leaves as a reach plant source of natural reducing compounds have gained recognition among the interests of researchers not only in terms of extraction but also in the synthesis of nanoparticles of various metals.^46–48^ Of all types of tea, green tea deserved special attention. AgNPs obtained through its extracts have been examined for antibacterial and antifungal activity.^45,49–55^ In certain cases, even their catalytic activity in the degradation of dyes present in industrial wastewater like Reactive Yellow 160,^56^ Direct Fast Rose FR Red 227,^57^ Basic Brilliant Flavine Y-40,^57^ Reactive Red 81,^58^ Congo Red Direct,^59^ Orange E3^60^ and Crystal Violet^61^ have been confirmed. Sun and his co-workers assessed the effects of time, optimal dosage of extract, and temperature on the synthesis of AgNPs (20–90 nm).^53^ The most pleasing result of synthesis was obtained after 2 h at room temperature. They also examined the inhibitory properties of these AgNPs against *Escherichia coli*. On the other hand, Babu et al. proposed an efficient method to synthesize these nanoparticles as a colorimetric sensor of cysteine.^52^ They employed different dilutions of the aqueous green tea extract under ambient temperature conditions. AgNPs possessed high stability for more than six months without the need for special storage conditions, such as low temperature or lack of light. Interestingly, it was also confirmed that the properties of AgNPs are significantly influenced by the elevation of the green tea growing fields in Nepal.^54^ More recently, Widatalla and coauthors demonstrated the high destructive activity of the produced AgNPs toward two bacteria: *Klebsiella pneumoniae* and *Staphylococcus aureus*.^62^ Onitsuka et al.^63^ compared the reducing properties of aqueous and methanolic extracts of green and black tea for the preparation of AgNPs. The stability of the AgNPs was comparable; however, a difference was observed in their size. For the aqueous extract, AgNPs were smaller for green tea, while for the methanolic extract using black tea leaves. Maintaining specified nanoparticle dimensions is necessary for commercializing AgNPs.^64^ Nucleation and agglomeration processes mainly influence the size of the synthesized nanoparticles. The model formation of nanoparticles assumes a two-step process: nucleation and growth.^65,66^ The course of these two stages is significantly influenced by process parameters such as pH value, temperature, ionic strength of the solution, and mixing intensity. Compounds in the reaction mixture, such as reducing and stabilizing agents, are equally important. It has been shown that trisodium citrate as a reducing agent and stabilizers such as sodium dodecyl sulfate (SDS) and Tween 80 influence the stability of AgNP suspension and prevent agglomeration.^67^ Biosynthesis using plant extract leads to nucleation and nanoparticle growth processes accompanied by numerous organic compounds whose effects are not yet fully understood.^68–70^

Despite the very rich literature based on the preparation of metallic nanoparticles using plant material as a source of potential reductants, there is still a discernible place to provide protocols related to optimizing efficient, sustainable, as well as green procedures in this research area. The ever-increasing industrial demand for nanomaterials that play an important role within the context of therapeutic, biotechnological, or technical applications is forcing the use of more friendly and inexpensive synthetic solutions based on the application of natural resources and decreasing the ecological footprint. The lack of one specific general extraction protocol tailored against all plants is currently a key problem facing the field of nanotechnology using plant extracts in the synthesis of nanomaterials. In this situation, scaling up nanosynthesis poses an additional challenge. This is due to the different conditions or regions of plant cultivation, the diversity in the profile of the chemical compounds hidden in them, and also the great flexibility of the parameters that determine effective extraction. The hope to deal with this problem is noticeable in process optimization. As mentioned above, the selection of specific parameters will make it possible to determine the conditions that ensure optimal effectivity by evaluating the relationship between them. Thus, it will be possible to reduce the economic effort and time required to develop the procedure. Therefore, keeping in mind the requirements of the industry, the study aimed to develop an efficient process for extracting polyphenolic compounds from ground green tea leaves based on three independent parameters: pH value, time, and extraction temperature, utilizing the combination prescribed by the statistical Box–Behnken response surface model. The total number of polyphenolic compounds (TPC) was used as a model response for two solvent systems with low environmental toxicity: water and ethanol–water mixture. Furthermore, an application test was conducted using the obtained extract under optimal conditions as a source of natural reducing agents for the biosynthesis of AgNPs.

## 2 Materials and Methods

### 2.1 Plant Material

Commercial store-bought green tea was used as the material for polyphenol extraction. Dried green tea leaves were ground using a domestic coffee grinder, thoroughly washed, and dried to increase the extraction efficiency. Each grinding process lasted for 1 min using the maximum power of the grinder. After the milling process, the particle size distribution of green tea was measured. The mean particle size was determined to be 205 μm using a Beckman Coulter LS 13 320 LS Particle Size Analyzer (see Figure S1).

### 2.2 Procedure for Polyphenol Extraction

The green tea leaves were mixed with an extraction solvent (0.2 g/25 mL) and left at room temperature before being heated to a specific temperature in a water bath. The biomass was then separated from the extract by filtration using a 0.22 μm filter (Sigma-Aldrich). The total amount of polyphenols in the extract was determined using the Folin–Ciocalteu method.^71–73^ Calibration curves for determining the total concentration of polyphenols in the aqueous and hydroalcoholic extracts are provided in the Supporting Information (see Supporting Information: Table S1 and Figure S2—for the aqueous extract, and Table S2 and Figure S3—for the hydroalcoholic). The final total polyphenol content was calculated using the equation shown below (eq 1)
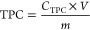
1

The elements of the equation represent the following: TPC [mg GAE/g db]—total polyphenol content, which is the response for statistical optimization; *C*_TPC_ [mg GAE/mL]—total polyphenol concentration calculated using the standard curve and accounting for the extract’s dilution; *V* [mL]—the volume of solvent selected for the extraction experiment; and *m*—the weight of the dry biomass (green tea leaves).

### 2.3 HPLC UV–Vis Analysis of Polyphenols in the Extract

HPLC UV–vis analyses were conducted to confirm the presence of characteristic components of tea in the aqueous and hydroethanolic extracts. A 500 μL sample of the extract was taken and diluted with the appropriate solvent by adding the same volume to the volumetric vial. The sample vial was placed in the measuring apparatus, an HPLC Dionex equipped with an Aeris PEPTIDE XB-C18 column (5 μm, 4.6 mm × 250 mm; Phenomenex Company). The mobile phase comprised solvents A (water with 0.1% TFA) and B (acetonitrile/water = 80/20, v/v). The measurement was performed using a gradient elution: 5–40% of solvent B for 30 min with UV detection at a wavelength of 280 nm. The injection volume was 20 μL, and the flow rate was 1.0 mL/min.

### 2.4 Procedure of AgNPs Biosynthesis

A flask equipped with a magnetic stirrer introduced an aqueous solution of silver nitrate (10^–3^ M). Then, 5 mL of the extract using the previously optimized conditions and a defined pH value were added dropwise. The mixture was heated in the dark at 65 °C for 45 min with constant stirring. The change in the color of the mixture and the appearance of light yellow turbidity indicated the reduction of Ag^+^ ions from the precursor to the Ag(0) nanoform. Once the reaction time had elapsed, the mixture was left to cool in the dark. The suspension of AgNPs was then transferred from the reaction vessel to an amber glass bottle and stored in a refrigerator at 4 °C for further characterization. A similar procedure for the synthesis of AgNPs using green tea extract and the temperature of 65 °C indicated as optimal, but with different proportions of the volume of the extract and the amount of AgNO_3_, was previously used by Riaz et al.^74^

### 2.5 Characterization of AgNPs Methods

The synthesized AgNPs were characterized using several techniques, including UV–vis spectra, DLS analysis, potential zeta, and TEM measurements.^43,55,75,76^ UV–vis spectral studies were conducted by using a Shimadzu UV–vis 1900i spectrophotometer with a typical plastic cuvette with an optical path of 10 mm. The measurement was taken 1 day after synthesis in the 200–800 nm wavelength range with simultaneous spectrum analysis using LabSolutions UV–vis software.^77^ The size of the AgNPs was analyzed using a diffraction light detector and a Photocor Complex analyzer (Photocor) at a temperature of 25 °C. TEM measurements were taken using an FEI Tecnai G2 20 X-TWIN high-resolution transmission electron microscope with a LaB6 cathode, an FEI Eagle 2K CCD camera, an EDS detector, and a STEM detector. The appropriate functional groups identifying the biomolecules present in the aqueous green tea extract and those resulting from distinct interactions between the polyphenolic compounds of the extract and the surface of the AgNPs formed with their participation as reducing agents were analyzed using Fourier transform infrared (FTIR) spectra. According to the standard pellet KBr method, FTIR measurements were performed for freshly prepared dried tea leaf extract as well as green tea extract-mediated AgNPs in the range of 4000 to 400 cm^–1^ using a Bruker Vertex 70 FTIR spectrometer.^50^

### 2.6 Box–Behnken Factorial Design for Extraction of Polyphenols from Green Tea L

The impact of three selected parameters, solvent pH, temperature (°C), and time (min), on the extracted total phenolic content was assessed using the Box–Behnken factorial design with three levels: (−1), (0), and (1). It should be emphasized here that temperature and time are the most important parameters factored in statistical designs in terms of optimizing the extraction of polyphenols from plant material, including tea leaves.^35,36,78^ The experimental design and levels for water and water–ethanol solvents are presented in Table 1, respectively.

**Table 1:** Three Optimization Parameters for the Extracting of Polyphenols from Green Tea Leaves Using Box–Behnken Factorial Design

independent variable (code units)	coded variable level			dependent variable (response)	goal
	(−1)	(0)	(1)		
Aqueous Extraction					
pH (*X*_1_)	4	5.5	7	total polyphenol content [mg GAE/g db] (TPC)	maximum
time [min] (*X*_2_)	10	20	30		
temperature [°C] (*X*_3_)	60	70	80		
Hydroethanolic Extraction					
pH (*X*_1_)	4	5.5	7	total polyphenol content [mg GAE/g db] (TPC)	maximum
time [min] (*X*_2_)	30	40	50		
temperature [°C] (*X*_3_)	60	70	80		

Based on the impact of different factor values on the extraction efficiency, which was expressed as TPC [mg GAE/g db], the levels of independent variables were determined and marked as (−1), (0), and (1) for statistical optimization using the Box–Behnken method. The coded values of the independent variables were calculated from their uncoded values using the following equation (eq 2), taking into account the process parameters.
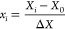
2where *x*_*i*_—is the mean dimensionless value of the process parameters, *X*_*i*_—is the uncoded value of the three test variables, *X*_0_—is the value of each uncoded variable at the central point, and Δ*x*—is the mean step change of the value of the actual variable (*X*_*i*_) corresponding to a variation of the coded value of process variables.

According to this design, 17 experiments were necessary to optimize and construct a model to generate the extraction efficiency expressed as the total polyphenol content (TPC) response. This included five replicated experiments under the same conditions where pH, temperature, and time were at level 0, as shown in Table 1, which served as the central reference. The three levels and range of values for the independent variables for water and the water–ethanol mixture are listed in Table 1, respectively. The experimental combinations of these variables following the selected statistical plan are presented for the aqueous extraction system in Table 2 and for the hydroethanolic extraction system in Table 3.

**Table 2:** Box–Behnken Factorial Design with Independent Variables and Experimental and Predicted TPC Values in Aqueous Green Tea Extract^a^

run	coded variable			actual variable			TPC [mg GAE/g db]		
	*x*_1_	*x*_2_ [min]	*x*_3_ [°C]	*X*_1_	*X*_2_ [min]	*X*_3_ [°C]	actual	predicted	residual
1	–1	–1	0	4.0	10	70	37.64	37.74	–0.10
2	1	–1	0	7.0	10	70	38.06	38.04	0.02
3	–1	1	0	4.0	30	70	38.14	38.16	–0.02
4	1	1	0	7.0	30	70	39.89	39.79	0.10
5	–1	0	–1	4.0	20	60	36.81	36.69	0.12
6	1	0	–1	7.0	20	60	38.06	38.06	0
7	–1	0	1	4.0	20	80	37.89	37.89	0
8	1	0	1	7.0	20	80	38.31	38.44	–0.13
9	0	–1	–1	5.5	10	60	37.64	37.66	–0.02
10	0	1	–1	5.5	30	60	39.23	39.33	–0.10
11	0	–1	1	5.5	10	80	39.14	39.04	0.10
12	0	1	1	5.5	30	80	39.56	39.54	0.02
13	0	0	0	5.5	20	70	37.89	37.59	0.30
14	0	0	0	5.5	20	70	37.64	37.59	0.05
15	0	0	0	5.5	20	70	37.48	37.59	–0.11
16	0	0	0	5.5	20	70	37.56	37.59	–0.03
17	0	0	0	5.5	20	70	37.39	37.59	–0.20

a *x*_1_ and *X*_1_—extraction solvent pH, *x*_2_ and *X*_2_—extraction time [min], *x*_3_ and *X*_3_—extraction temperature [°C].

**Table 3:** Results of ANOVA Analysis for the Quadratic Regression Model of the TPC in the Aqueous Extraction of Green Tea L^a^

interaction type	source	TPC [mg GAE/g db]					
		coefficient factor	SS	df	MS	*F*-value	*p*-value
	model	37.592	11.06617	9	1.22957	38.9845	<0.0001*
linear	*x*_1_	0.480	1.84320	1	1.84320	50.6791	0.0021*
	*x*_2_	0.543	2.35445	1	2.35445	64.7360	0.0013*
	*x*_3_	0.395	1.24820	1	1.24820	34.3195	0.0042*
quadratic	*x*_1_^2^	–0.142	0.08520	1	0.08520	2.3426	0.2006
	*x*_2_^2^	0.983	4.06652	1	4.06652	111.8096	0.0005*
	*x*_3_^2^	0.318	0.42512	1	0.42512	11.6886	0.0268*
interaction	*x*_1_*x*_2_	0.333	0.44223	1	0.44223	12.1591	0.0252*
	*x*_1_*x*_3_	–0.208	0.17222	1	0.17222	4.7354	0.0952
	*x*_2_*x*_3_	–0.293	0.34222	1	0.34222	9.4095	0.0374*
residual			0.22078	7	0.03154		
pure error			0.14548	4	0.03637		
lack of fit			0.07530	3	0.02510	0.6901	0.6039
core total			11.28695	16			

a SD = 0.84; mean = 38.14; *R*^2^ = 0.9804; *R*_adj_^2^ = 0.9553; *R*_pred_^2^ = 0.9807; C.V. % = 2.2; PRESS = 0.2184; adequate precision = 23.2733; SS—sum of squares; MS—mean square; PRESS—predicted residual error sum of squares; df—degree of freedom * significant for *p* < 0.05

The second-order polynomial equation, which is the image of the response function obtained by regression analysis, is presented by eq 3
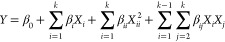
3where *Y*—is the predicted response (total polyphenol content—TPC [mg GAE/g db]), β_0_, β_*i*_, and β_*ii*_, β_*ij*_—are regression coefficients, which represent the variable correlation response: β_*i*_—is the linear coefficients, β_*ii*_—is the quadratic coefficients, and β_*ij*_—is the coefficients of cross-interactions between two independent variables, *k*—is the number of analyzed independent variables, *X*_*i*_ and *X*_*j*_—are the values of the independent variables.

The statistical calculation software Statistica 13.3 was utilized to perform the regression analysis and obtain the coefficients of the polynomial equation presented above. The ANOVA statistical test provided by the program allowed for the correction of the regression equation by assessing the model’s usefulness using parameters such as lack of fit (LoF) and *p*-value, *R*^2^ and *R*_adj_^2^ coefficients, and obtaining the response surface curvature.

## 3 Results and Discussion

**Figure 1:**
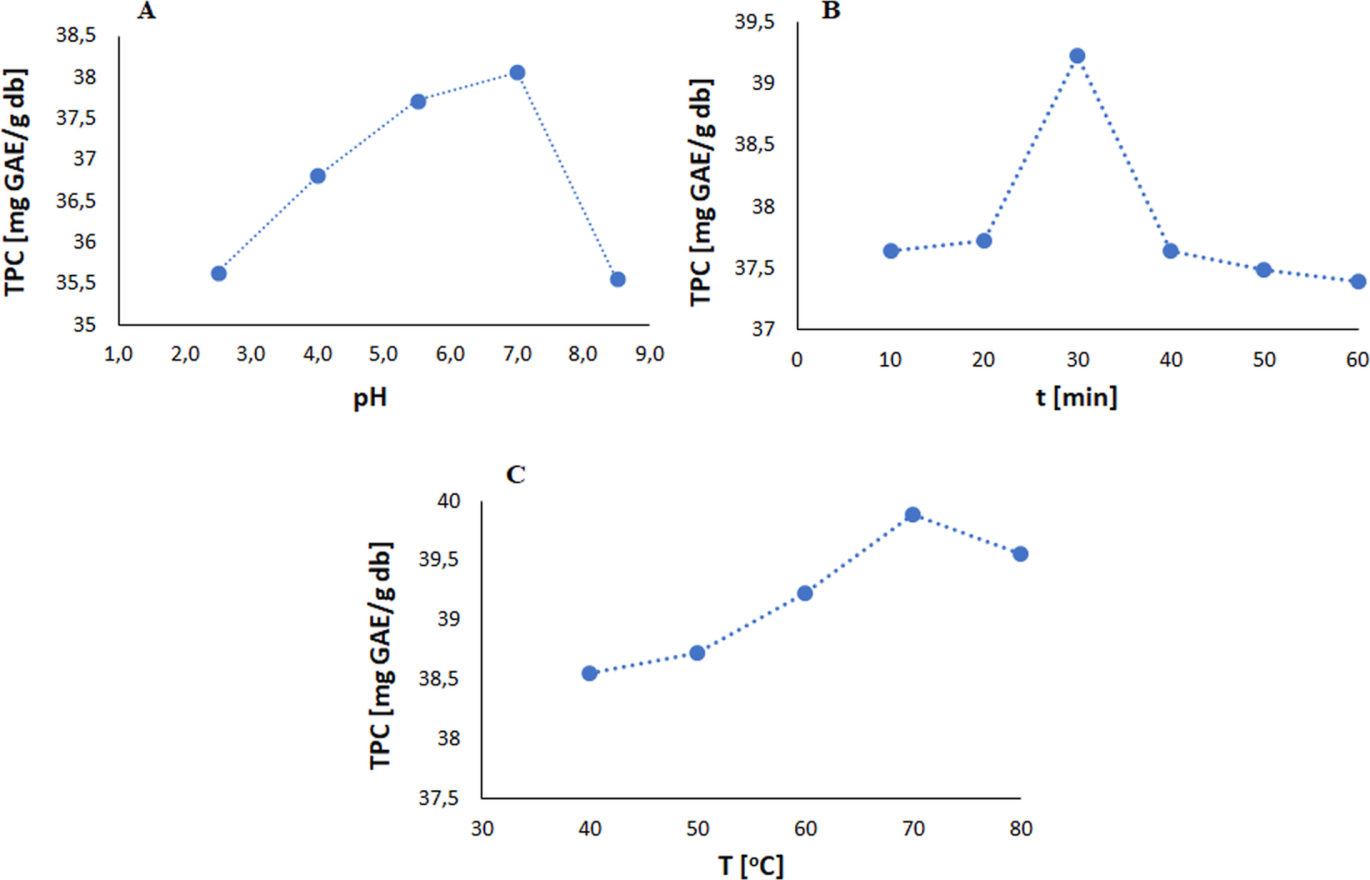
Single-factor experiments: (A) pH, (B) time, and (C) temperature effects on the aqueous polyphenol extraction from Green Tea L.

**Figure 2:**
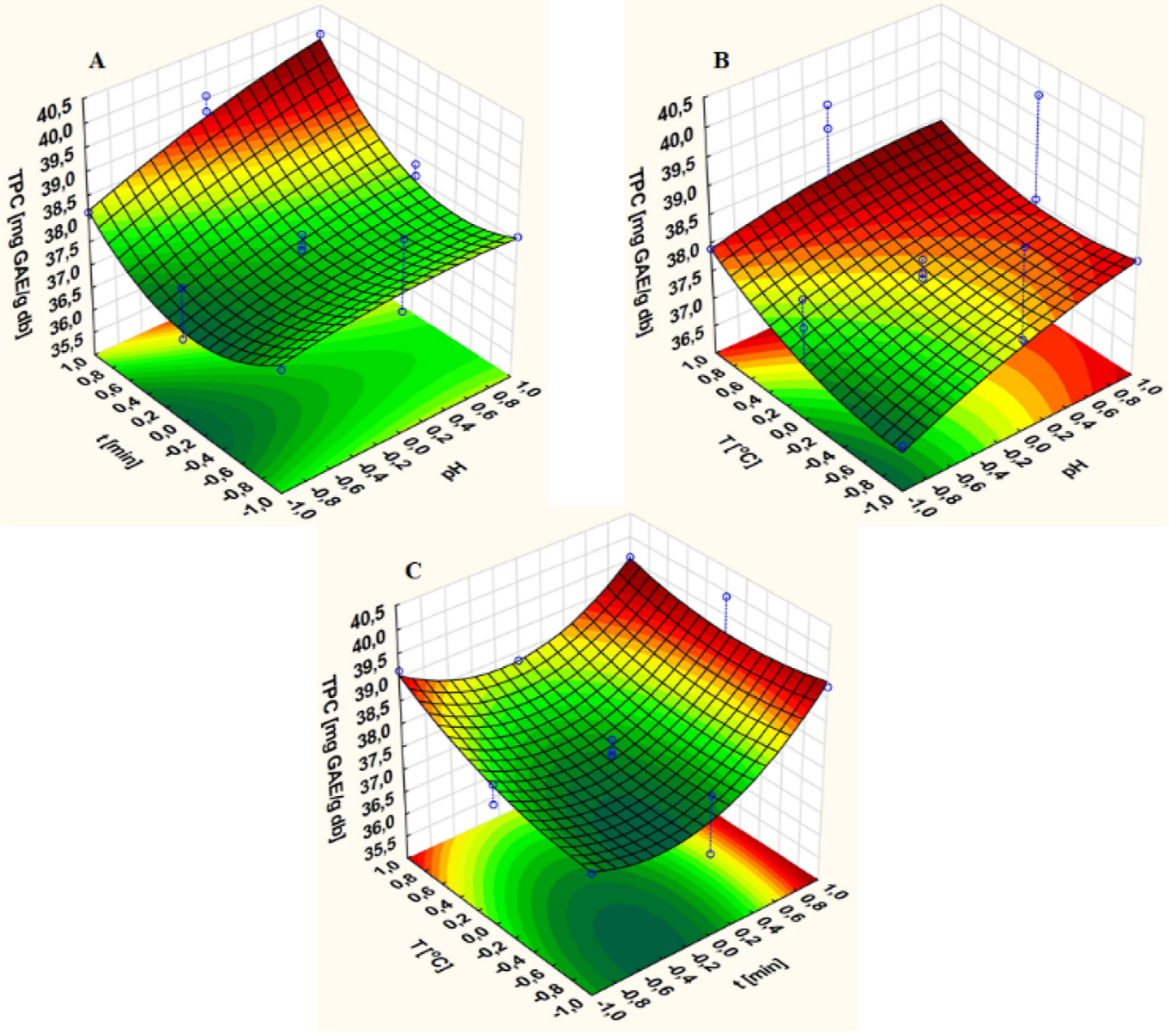
Response surface 3D plots for the result of the interaction effect between (A) pH value vs time, (B) pH value vs temperature, and (C) time vs temperature on the TPC yield in water.

The green synthesis of AgNPs using plant extracts is a common method for obtaining metallic nanoparticles while meeting the principles of green chemistry. Many reports in the literature, developed over more than a hundred years, have concluded that several factors affect the efficiency and course of the extraction process of bioactive compounds from plant structural elements. Carrying out experiments to determine the optimal value for each factor is an impractical point of view. Therefore, an essential step in developing an effective green technological extraction or AgNP formation process is to select a few parameters and conduct preliminary experiments to determine their most effective values. Based on literature data, the parameters selected for optimizing the polyphenol extraction process from Green Tea L. are pH, extraction process duration, and temperature (Figures 1 and 2).

### 3.1 Effect of Single Parameter Value on the Total Polyphenol Content of Aqueous Extraction of Green Tea L

An initial step in the work was to study the effect of individual parameters on the extraction process of polyphenols from Green Tea L. in water (Figure 1). Figure 1 shows the results of three successive series of factorial experiments for the extraction of Green Tea L. and their influence on the efficiency of the extraction process expressed in the form of TPC [mg GAE/g db]: total polyphenol content. The ranges for these parameters were as follows: a pH value of 2.5 to 8.5 (Figure 1A), an extraction time of 10 to 60 min (Figure 1B), and an extraction temperature of 40–80 °C (Figure 1C).

The first parameter analyzed for the extraction of polyphenols from green tea leaves was the pH value of the water, while the other parameters were kept constant (20 min and 60 °C) (Figure 1A). The results showed that as the pH increased from acidic to neutral, the extraction efficiency increased, reaching its maximum value of 38.06 mg of GAE/g of db at a neutral pH. This observation is consistent with the trend observed in the extraction with tea, where polyphenolic compounds were found to be more stable in an acidic environment than in an alkaline environment. Conversely, using a pH in the alkaline range could lead to conformational changes, causing the appearance of decomposition products of polyphenolic compounds, resulting in a reduction or complete loss of their amount.^79^ This explains why the extraction efficiency decreases when the water’s pH is beyond the neutral value. The analysis of extraction yields (TPC) at different time intervals showed that initially, the extraction efficiency increased and reached its maximal value after 30 min (Figure 1B). However, when the extraction process was extended beyond 40 to 60 min, there was a sluggish decrease in the extraction efficiency, which could be due to the decomposition of polyphenols into compounds of lower molecular weight of prolonged heating at a given temperature. The final set of experiments focused on temperature, which is considered one of the most important extraction parameters,^78,80^ within 40 to 80 °C (Figure 1C). The results showed that an increase in the extraction temperature led to an increase in efficiency.

The highest TPC value was recorded for the temperature at 70 °C, reaching 39.89 [mg GAE/g db]. This finding is consistent with the general statement that traditional methods of polyphenol extraction achieve the highest level of isolation at temperatures between 60 and 80 °C.^81^ However, there was a slight decrease in extraction efficiency when the temperature was increased to 80 °C, possibly due to the formation of insoluble forms of polyphenolic compounds as a result of their detachment from the cell wall or decomposition processes of the entire plant cell.^80,82,83^ The final values of the independent variables pH (4, 5.5, and 7), time (10, 20, and 30 min), and temperature (60, 70, and 80 °C) used for statistical modeling for water extraction, selected based on the described single-factor experiments, are summarized in Table 1.

### 3.2 Box–Behnken Design Optimization of the Green Tea L. Extraction Conditions in an Aqueous System and RSM Analysis

Optimizing the polyphenol extraction process was based using the Box–Behnken statistical model and analyzed using the response surface methodology (RSM). Three independent variables, including pH value, time, and temperature, were assessed according to the planned experiment. The Box–Behnken model matrix, with imposed connections between the extracted parameters’ coded and uncoded values, was used to design 17 experiments, as shown in Table 2. The table includes each efficiency value (TPC [mg GAE/g db]) for each extraction experiment’s efficiency value (TPC [mg GAE/g db]).

The experimental data for the extraction of Green Tea L. showed that the yields (TPC) of this process ranged from 37.39 to 39.89 [mg of GAE/g db]. The highest TPC value was obtained by performing the extraction at 70 °C for 30 min while maintaining a neutral pH of water as the solvent. The lowest yield was obtained when the sample was heated at the same temperature for 20 min in a solvent with an acidic pH value of 5.5. Based on all results, the average extraction efficiency of polyphenolic compounds in water was 38.14 [mg of GAE/g db].

The prediction model for the extraction of polyphenols from dried Green Tea L. in an aqueous environment is described by the following equation (eq 4)
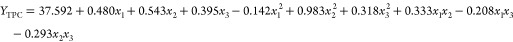
4where *x*_1_, *x*_2_, and *x*_3_ denote the coded independent variables: pH value, time, and temperature, respectively. The model and, thus, its final equation were verified by statistical analysis, which was the ANOVA test, and its results are presented in Table 3.

Examination of the parameters obtained through the ANOVA statistical test allowed for assessing the significance of the generated mathematical model for extracting polyphenols from Green Tea L. and identifying the factor that had the greatest impact on the course of the process (Table 3, see Supporting Information: Figure S4). The probability value (*p*-value) is used to estimate the significance of the model. Typically, the response variable is considered significant if the *p*-value is less than 0.05.

In the tested model for the effective extraction of polyphenols from green tea leaves, all the generated linear coefficients for the variables pH (*x*_1_), time (*x*_2_), and temperature (*x*_3_) were found to be significant, as well as the quadratic coefficients related to time (*x*_2_^2^) and temperature (*x*_3_^2^) variables, and the interaction coefficients between the pH value and time (*x*_1_*x*_2_) and time and temperature of extraction (*x*_2_*x*_3_). However, the square coefficient of pH (*x*_1_^2^) and the interaction between pH and temperature (*x*_1_*x*_3_) were insignificant. After the insignificant terms are removed from the model, the final equation describing the investigated response of the process is presented in the form of eq 5
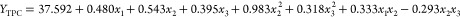
5

Other parameters such as the correlation coefficient (*R*^2^), adjusted correlation coefficient (*R*_adj_^2^), predicted coefficient (*R*_pred_^2^), F-value of the model and LoF significance, adequate precision, and coefficient of variation (C.V.) are crucial for validating the obtained model. The prepared regression analysis yielded an *R*^2^ correlation coefficient of 0.9804, indicating a good fit between the experimental and model-predicted extraction yields expressed as TPC. The linear fit is shown in Figure S5 (see Supporting Information). The equally high value of the adjusted *R*^2^, which is 0.9553, provides information about the model’s ability to explain 95.53% of all variances. The predicted *R*^2^ (0.9807) is close to *R*_adj_^2^, which is desirable, as they should agree and the difference between them should be less than 0.2. The model’s usefulness is further demonstrated by the insignificant value of the LoF (>0.05) and the significant *p*-value for the *F*-value of the model, which is less than 0.0001. Furthermore, the C.V. parameter value of 2.2%, which is lower than 10, confirms the model’s accuracy. The estimated value of the signal-to-noise ratio, equal to 23.2733, also called adequate precision, exceeds the value of 4, which additionally indicates that the obtained model is precise in determining the optimal conditions based on the analysis of the surface response in the form of three-dimensional (3D) graphs (Figure 2).

These graphs illustrate the interactions between the extraction parameters, such as the pH value of water and time (Figure 2A), the pH value of water and temperature (Figure 2B), and the interaction between time and temperature (Figure 2C). The mutual interaction between the pH value and time has the greatest impact on the extraction process.

Based on the 3D plots of the surface response, the optimal conditions for the extraction of polyphenols from Green Tea L. were identified, with the highest yield of 40 [mg GAE/g db] achieved at pH = 7, 30 min and 80 °C (see Supporting Information: Figure S8). Laboratory experiments confirmed the compatibility between predicted and experimental TPC values under optimal conditions, with an average value of 40.34 [mg GAE/g db] in three replicates.

### 3.3 Effect of Single Parameter Value on the Total Polyphenol Content of Hydroethanolic Extraction of Green Tea L

After water, alcohols have become successful group of solvents for extracting various bioactive compounds from plants.^84–87^ They are used either as an absolute alcohol or as a mixture of water. Additionally, because of their low boiling point, it is easy to evaporate them, thereby obtaining pure bioactive compounds that can be further analyzed.

**Figure 3:**
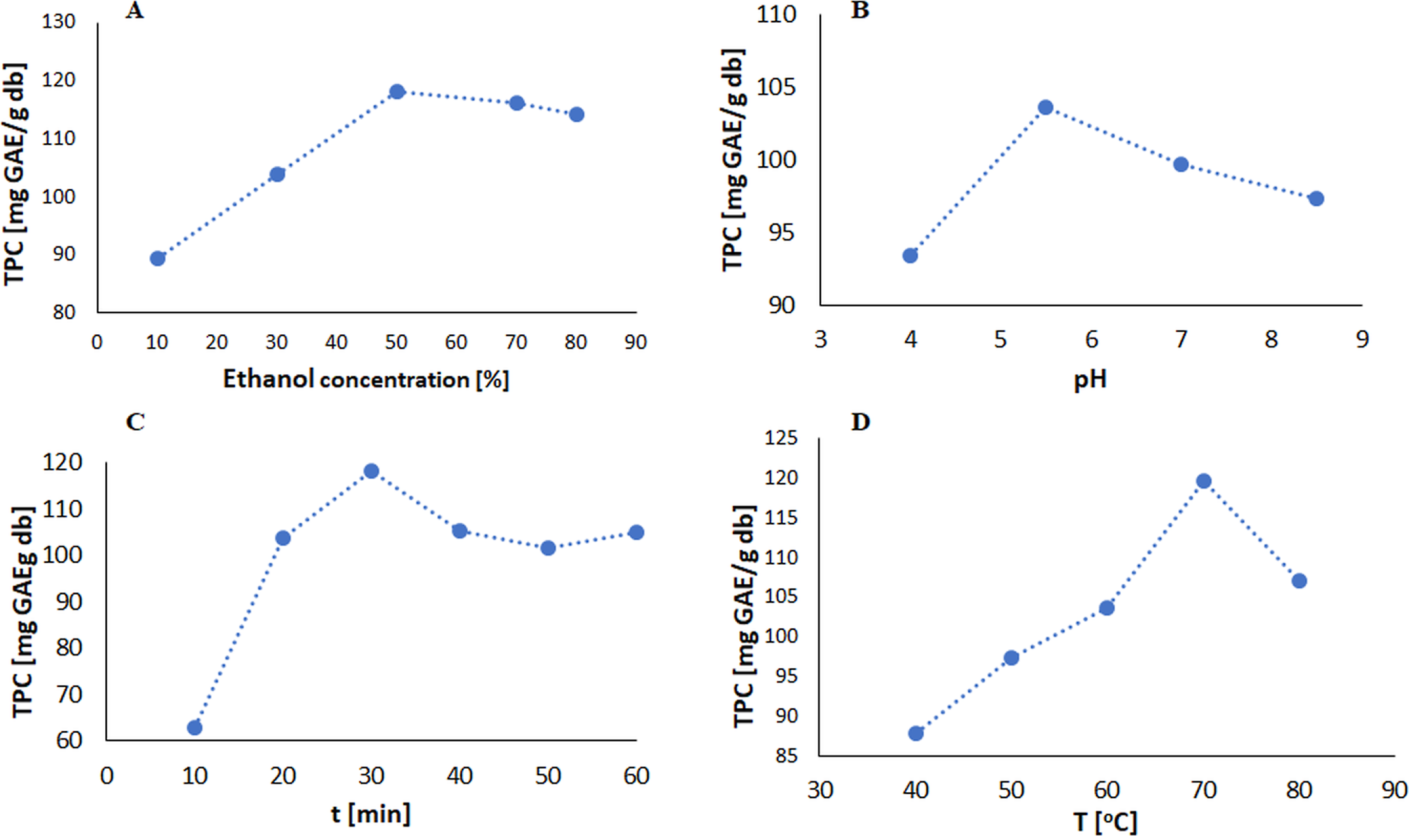
Single-factor experiments: (A) ethanol concentration, (B) pH, (C) time, and (D) temperature effect on the hydroethanolic polyphenol extraction from Green Tea L.

The optimal composition of an ethanol and water mixture as an extractant for the extraction of green tea leaves was determined first (Figure 3). Figure 3A shows that the range of ethanol concentrations used for obtaining the most effective extraction of ground and dried leaves was between 10 and 80%. After selecting an appropriate ethanol/water ratio mixture of 1:1, a series of single-factor experiments based on pH (4–8.5), time (10–60 min), and temperature (40–80 °C) were conducted similar to the water extraction process. The results of these are presented in Figure 3B–D.

The extraction efficiency in the ethanol/water mixture increased with increasing concentration up to 50%, which provided the optimal TPC value of about 118 mg of GAE/g of db (Figure 3A). At higher concentrations, a gradual reduction in the amount of polyphenols in the hydroalcoholic extract was observed, which was possible due to the destruction of cells in the plant material, which can hinder the isolation of desired bioactive compounds.^88^

Three levels of extraction parameters—pH, time, and temperature—were selected based on the results of single-factor parameter experiments (Table 1 and Figure 3). Extraction efficiency increased to 103.64 mg of GAE/g of db in the acidic pH range and peaked at pH 5.5 (Figure 3B). In contrast, extraction performed at higher pH values led to a gradual reduction in the yield of polyphenolic substances, which is consistent with the previously presented theory regarding the transformation of the conformational structure of polyphenols and their subsequent degradation and decay. Based on these results, the same pH levels used in the water system (−1) 4, (0) 5.5, and (1) 7 were selected for RSM studies.

The next part of the experiments focused on optimizing the extraction time in the hydroalcoholic system (Figure 3C). Like the water system, the maximum extraction yield of 118.13 mg of GAE/g of db was obtained after 30 min. Extending the extraction time to 40 min slightly decreased the efficiency of the process, which remained relatively constant in subsequent min. The extraction time factor levels chosen for the statistical model were: (−1) 30 min, (0) 40 min, and (1) 50 min. It is worth noting that these levels differed from those used in water extraction.

The temperature was the last parameter optimized in the single-factor experiments (Figure 3D). Like the aqueous system, the efficiency of obtaining polyphenols in the hydroalcoholic system increased with an increase in temperature at 40–70 °C, reaching the maximum value of 119.73 [mg GAE/g db]. However, at 80 °C, there was a slight decrease in the extraction yield. The following temperature factor levels were used for statistical experiments: (−1) 60 °C, (0) 70 °C, and (1) 80 °C.

### 3.4 Box–Behnken Design Optimization of the Green Tea L. Extraction Conditions in Hydroethanolic System and RSM Analysis

The polyphenol extraction process from green tea leaves using a 50% ethanol–water solution was optimized using the Box–Behnken mathematical design, similar to water extraction. The matrix for this design consisted of 17 experiments, and the parameter values and results obtained are shown in Table 4.

**Table 4:** Box–Behnken Factorial Design with Independent Variables and Experimental and Predicted TPC Values in Hydroethanolic Green Tea L. Extract^a^

run	coded variable			actual variable			TPC [mg GAE/g db]		
	*x*_1_	*x*_2_ [min]	*x*_3_ [°C]	*X*_1_	*X*_2_ [min]	*X*_3_ [°C]	actual	predicted	residual
1	–1	–1	0	4	30	70	108.75	110.17	–1.42
2	1	–1	0	7	30	70	110.45	109.81	0.64
3	–1	1	0	4	50	70	109.89	110.53	–0.64
4	1	1	0	7	50	70	114.15	112.73	1.42
5	–1	0	–1	4	40	60	107.61	106.80	0.81
6	1	0	–1	7	40	60	109.31	110.56	–1.25
7	–1	0	1	4	40	80	114.15	112.91	1.24
8	1	0	1	7	40	80	110.18	110.99	–0.81
9	0	–1	–1	5.5	30	60	118.13	117.52	0.61
10	0	1	–1	5.5	50	60	113.86	114.04	–0.18
11	0	–1	1	5.5	30	80	115.85	115.67	0.18
12	0	1	1	5.5	50	80	121.82	122.43	–0.61
13	0	0	0	5.5	40	70	118.69	119.71	–1.02
14	0	0	0	5.5	40	70	120.40	119.71	0.69
15	0	0	0	5.5	40	70	120.11	119.71	0.40
16	0	0	0	5.5	40	70	120.40	119.71	0.69
17	0	0	0	5.5	40	70	118.97	119.71	–0.74

a *x*_1_ and *X*_1_—extraction solvent pH, *x*_2_ and *X*_2_—extraction time [min], *x*_3_ and *X*_3_—extraction temperature [°C]

Table 4 presents the uncoded and coded values of the parameters representing the Box–Behnken matrix used to describe the extraction of Green Tea L. using an ethanol/water mixture and the results of the process efficiencies marked as TPC. Based on the data, it can be concluded that extraction in the hydroalcoholic system is much more effective than extraction in an aqueous solvent, with TPC values up to three times higher.

The extraction efficiency ranged from 107.61 to 121.82 [mg of GAE/g db]. The highest extraction efficiency was achieved when isolating polyphenols at 80 °C for 50 min with a constant pH of the solvent of 5.5, while the lowest value was obtained for the pH value of the ethanol/water mixture equal to 4.0 during 40 min at 60 °C. The average TPC value calculated from all extraction results was 114.86 [mg GAE/g db].

After the necessary data were collected, a linear regression analysis was performed to obtain an equation describing the optimal model (eq 6). This equation can be used to determine the predicted TPC values under indicated conditions, including the optimal conditions, which can then be compared with experimental values to check the reliability of the model.
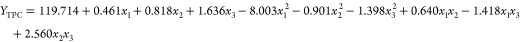
6

In the equation, *x*_1_, *x*_2_, and *x*_3_ represent independent variables in the coded form: pH value, time, and temperature, respectively.

The significance of the green tea extraction model using an aqueous ethanol solvent was determined based on the ANOVA test results shown in Table 5, considering the *p*-value while simultaneously determining the quadratic correlation of the pH factor.

**Table 5:** Results of ANOVA Analysis for the Quadratic Regression Model of the TPC in the Hydroethanolic Extraction of Polyphenols^a^

interaction type	source	TPC [mg GAE/g db]					
		coefficient factor	SS	df	MS	*F*-value	*p*-value
	model	119.7140	356.6038	9	39.6226	21.7134	<0.0001*
linear	*x*_1_	0.4613	1.7020	1	1.7020	2.5212	0.1875
	*x*_2_	0.8175	5.3465	1	5.3465	7.9203	0.0481*
	*x*_3_	1.6363	21.4185	1	21.4185	31.7297	0.0049*
quadratic	*x*_1_^2^	–8.0032	269.6927	1	269.6927	399.5269	<0.0001*
	*x*_2_^2^	–0.9007	3.4162	1	3.4162	5.0608	0.0877
	*x*_3_^2^	–1.3982	8.2320	1	8.2320	12.1950	0.0251*
interaction	*x*_1_*x*_2_	0.6400	1.6384	1	1.6384	2.4272	0.1942
	*x*_1_*x*_3_	–1.4175	8.0372	1	8.0372	11.9065	0.0260*
	*x*_2_*x*_3_	2.5600	26.2144	1	26.2144	38.8344	0.0034*
residual			12.7736	7	1.8248		
pure error			2.7001	4	0.6750		
lack of fit			10.0732	3	3.3577		0.0776
core total			369.3774	16			

a SD = 4.80; mean = 114.87; *R*^2^ = 0.9654; *R*_adj_^2^ = 0.9210; *R*_pred_^2^ = 0.9654; C.V. % = 4.2; PRESS = 12.7735; adequate precision = 15.4279; SS—sum of squares; MS—mean square; PRESS—predicted residual error sum of squares; df—degree of freedom * significant for *p* < 0.05

This factor strongly influences, as confirmed by the Pareto graph (see Supporting Information: Figure S6). Significant effects observed include linear correlations of time (*x*_2_) and temperature (*x*_3_), quadratic correlations described by coefficients of pH (*x*_1_^2^) and temperature (*x*_3_^2^), and coefficients describing the interaction between pH and extraction temperature (*x*_1_*x*_3_) as well as time and temperature (*x*_2_*x*_3_). Conversely, coefficients calculated for the linear pH effect (*x*_1_), the quadratic time coefficient (*x*_2_^2^), and the coefficient calculated from the interaction between pH and time (*x*_1_*x*_2_) were insignificant. Thus, they should be omitted in the final extraction model equation, as represented by eq 7
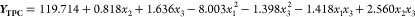
7

**Figure 4:**
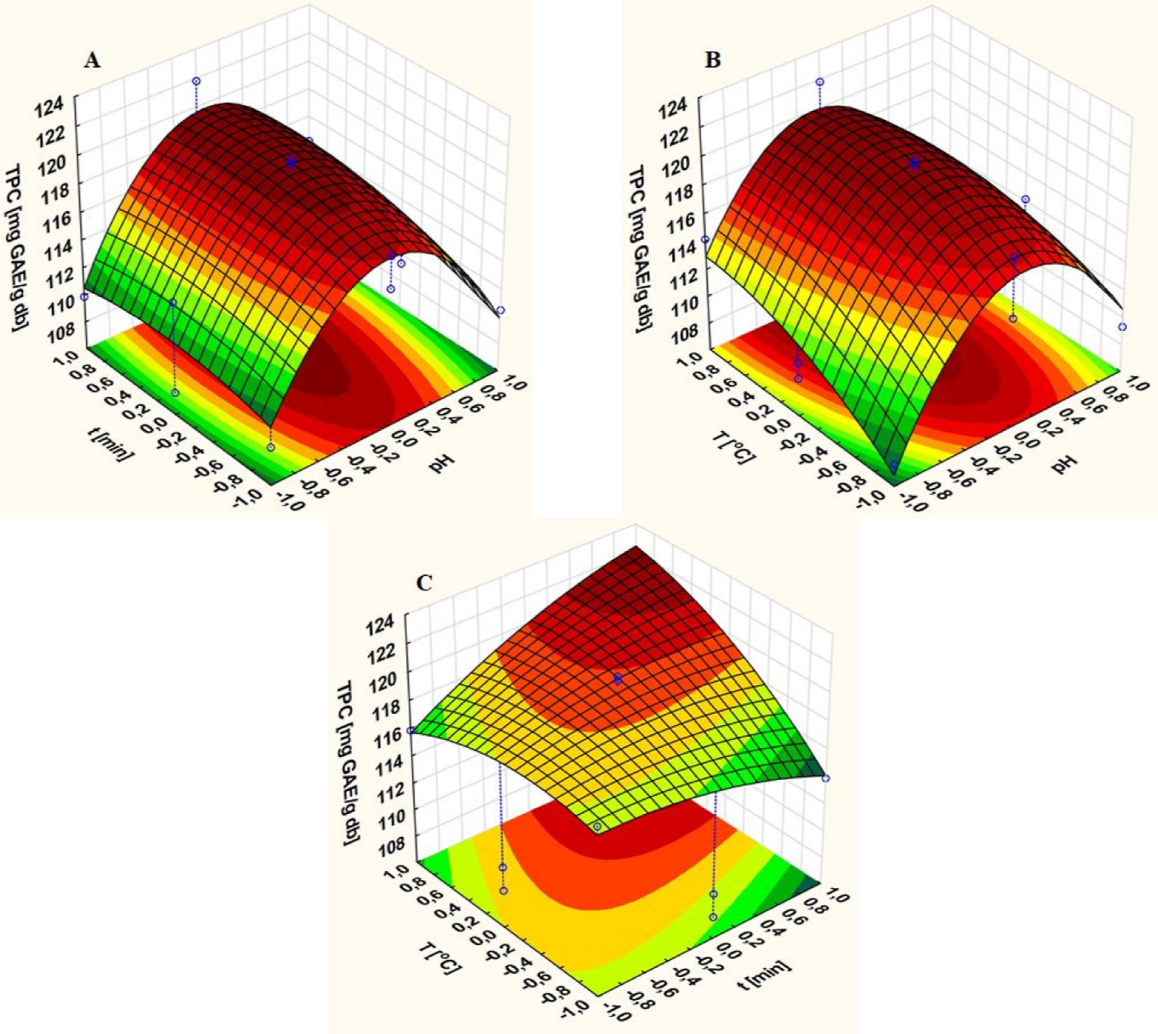
Response surface 3D plots for the result of an interaction effect between (A) pH value vs time, (B) pH value vs temperature, and (C) time vs temperature on the TPC yield in an ethanol/water mixture.

The model’s usefulness was also evaluated regarding other statistical parameters deemed suitable. These include coefficients such as *R*^2^, *R*_adj_^2^, and *R*_pred_^2^, the *F*-value of the model, LoF significance, adequate precision, and the coefficient of variation (C.V.). The *R*^2^, *R*_adj_^2^, and *R*_pred_^2^ coefficients are satisfactory, exceeding 0.9 and equaling 0.9654, 0.9210, and 0.9654, respectively. This indicates that the model effectively describes 92.10% of all variances represented by the model, and the data values predicted by the model closely match the experimental data concerning TPC obtained in the hydroethanolic extraction process as observed in the aqueous system. The highly linear relationship between the data was further confirmed by the comparison shown in Figure S7 (see Supporting Information). The insignificance of the LoF parameter of 0.0776 and the highly significant *p*-value parameter for the entire model (less than 0.0001) allow the model to be accepted as useful for determining optimal conditions for extracting polyphenols from green tea leaves using an ethanol/water mixture. For this model, the adequate precision and coefficient of variation values were determined to be 15.4279 and 4.2, respectively, falling within the range established by statistical rules. This led to the full characterization of the model and the possibility of using response surface analysis to determine the optimal extraction conditions (Figure 4).

According to the three-dimensional graphs obtained by modeling (Figure 4A–C), which show the interrelationships between two selected extraction parameters, the pH value of the ethanol/water mixture and time, the pH value of the solvent mixture and temperature, and time and temperature values, while maintaining a constant value of the third parameter, the interaction between time and temperature has the strongest influence on the extraction process. The optimal conditions for this process were determined to be a pH of 5.5, a duration of 50 min, and a temperature of 80 °C (see Supporting Information: Figure S9).

This combination of results appears in the Box–Behnken model matrix and represents the highest extraction process efficiency, 121.82 [mg of GAE/g db]. The TPC value predicted by the model is 122.43 mg of GAE/g of db, indicating that both values are very close. Based on this result, it can be concluded that the proposed model is a good fit and can be used to determine the TPC value for extracting the selected plant research material.

### 3.5 Characterization of AgNPs

**Figure 5:**
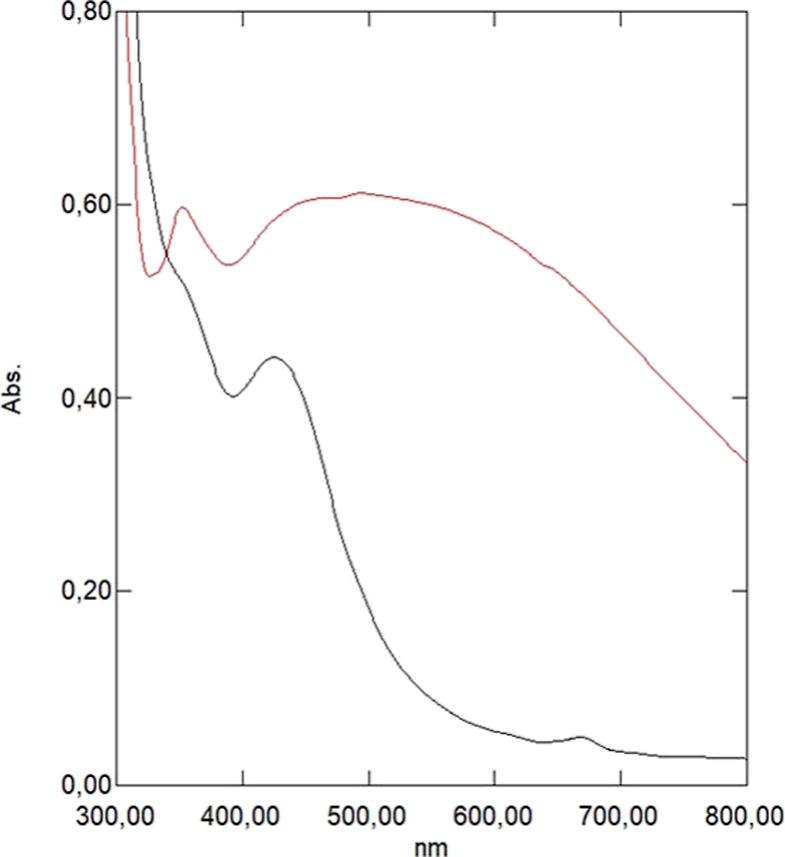
UV–vis spectra of AgNPs synthesized using aqueous (red) and hydroethanolic (black) extracts of Green Tea L.

The first evidence for the biosynthesis of AgNPs using an aqueous or hydroalcoholic extract of Green Tea L. was a visible change in the color of the reaction mixture from colorless to a yellow-green or light brown suspension, depending on the extract used. Additionally, spectrophotometric studies conducted in the subsequent step confirmed this fact by identifying the maximum surface plasmon resonance absorbance found in the typical wavelength range for AgNPs, between 420 and 500 nm (Figure 5).

**Figure 6:**
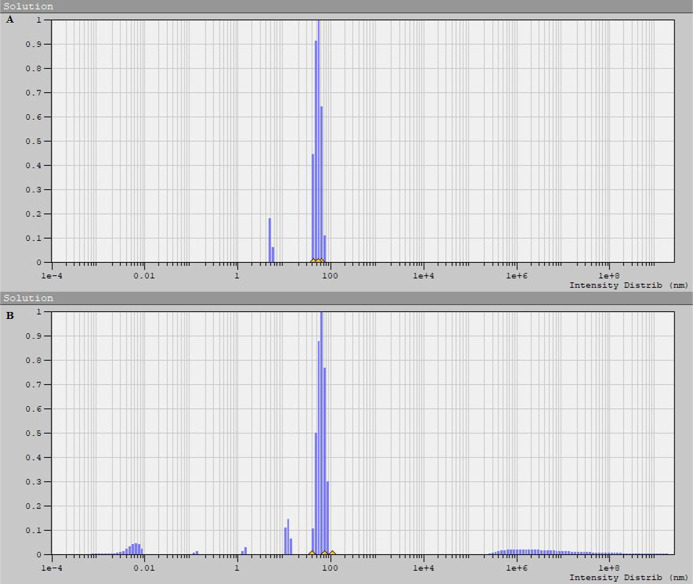
Distribution of radii of AgNPs obtained using aqueous (A) and hydroalcoholic (B) extracts.

Notably, the AgNPs obtained using both proposed plant extracts as natural reducing agents exhibit differences in morphology primarily due to their size (Figure 6).

**Figure 7:**
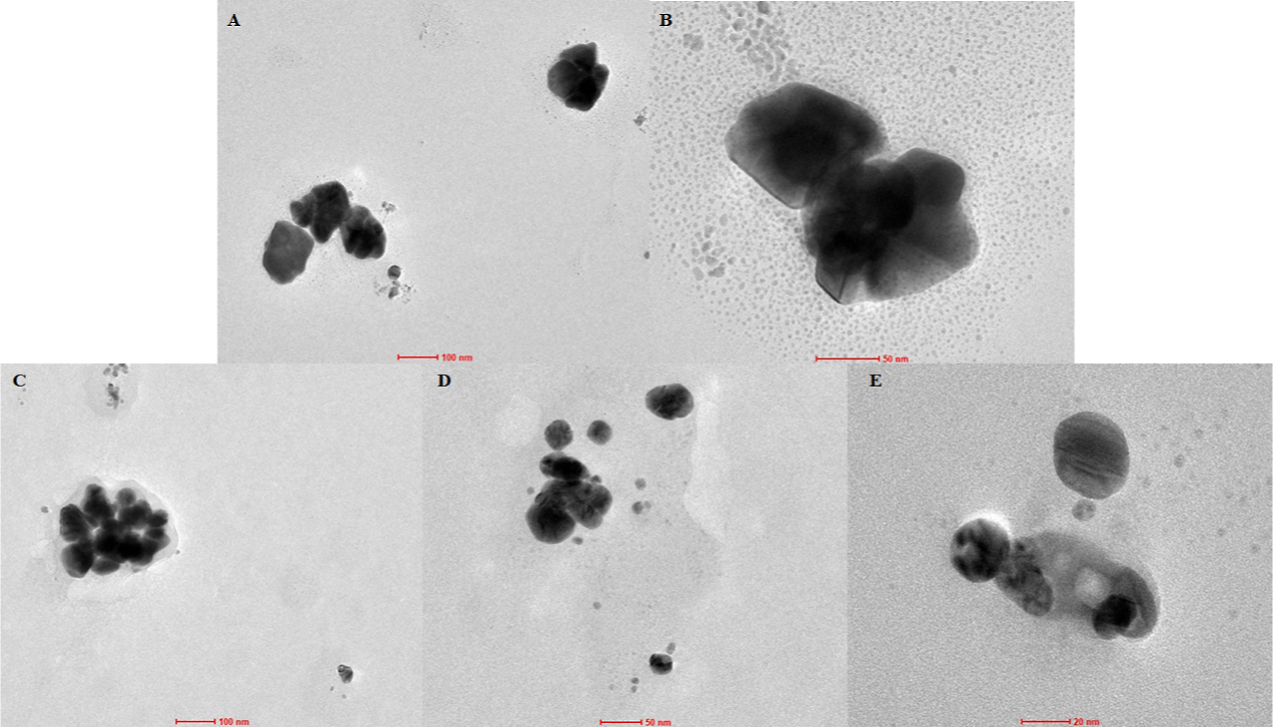
TEM images of AgNPs obtained using (A,B) aqueous extract and (C–E) hydroalcoholic extract

AgNPs formed during the green synthesis, where the hydroethanolic extract was employed, showed a tendency to exist in large aggregates, with an average size of nanoparticles equal to 139.8 nm (Figures 6B and 7C–E), while in the case of the aqueous extract it was 101.99 nm (Figures 6A and 7A,B).

The reason for their ability to agglomerate should be sought in the qualitative and quantitative composition of both extracts used for the synthesis (see Supporting Information: Table S3). The composition of the main polyphenols found in the aqueous and hydroethanolic extracts indicates that a higher amount of them is achieved using an ethanol/water mixture for the extraction process. This is in agreement with the results of ethanol extraction reported in the literature.^89^

As per the literature,^90^ the nucleation and aggregation of nanoparticles are strongly related to the bioreduction substances present in the extracts. When an ethanol/water mixture is used, a higher quantity of extracted substances can cause the aggregation of AgNPs. This is due to a decrease in the surface tension of the reaction solution.^91,92^

The reducing properties of ethanol in the synthesis of metal nanoparticles have been described in the literature.^93^ Therefore, an appropriate experiment was carried out to confirm the effect of 50% ethanol on the course of AgNP synthesis. Reduction of the silver precursor with 5 mL of a water/ethanol (1:1) mixture that replaced the plant extract as reducing agent failed under the same reaction conditions. The peak confirming the formation of AgNPs did not appear in the UV–vis spectra either immediately after the reduction process or after 24 h of storage of the sample, which was protected from the light. It can therefore be assumed that the ratio of the water/ethanol mixture used in the extract increases the efficiency of polyphenol extraction compared to water but has no direct effect on the synthesis of AgNPs. Probably, the concentration of ethanol and its amount may be insufficient to observe the symptoms of reduction in the conditions of synthesis proposed by us. It provides information that only biomolecules present in the extract are responsible for the reduction process.

Additionally, it should be emphasized that (−)epigallocatechin gallate (EGCG) and (−)epicatechin-3-gallate (ECG), which were found in higher concentrations in the hydroethanolic extract compared to the aqueous extract, may have the most significant impact on the reduction of the silver precursor to nanoparticles.

**Figure 8:**
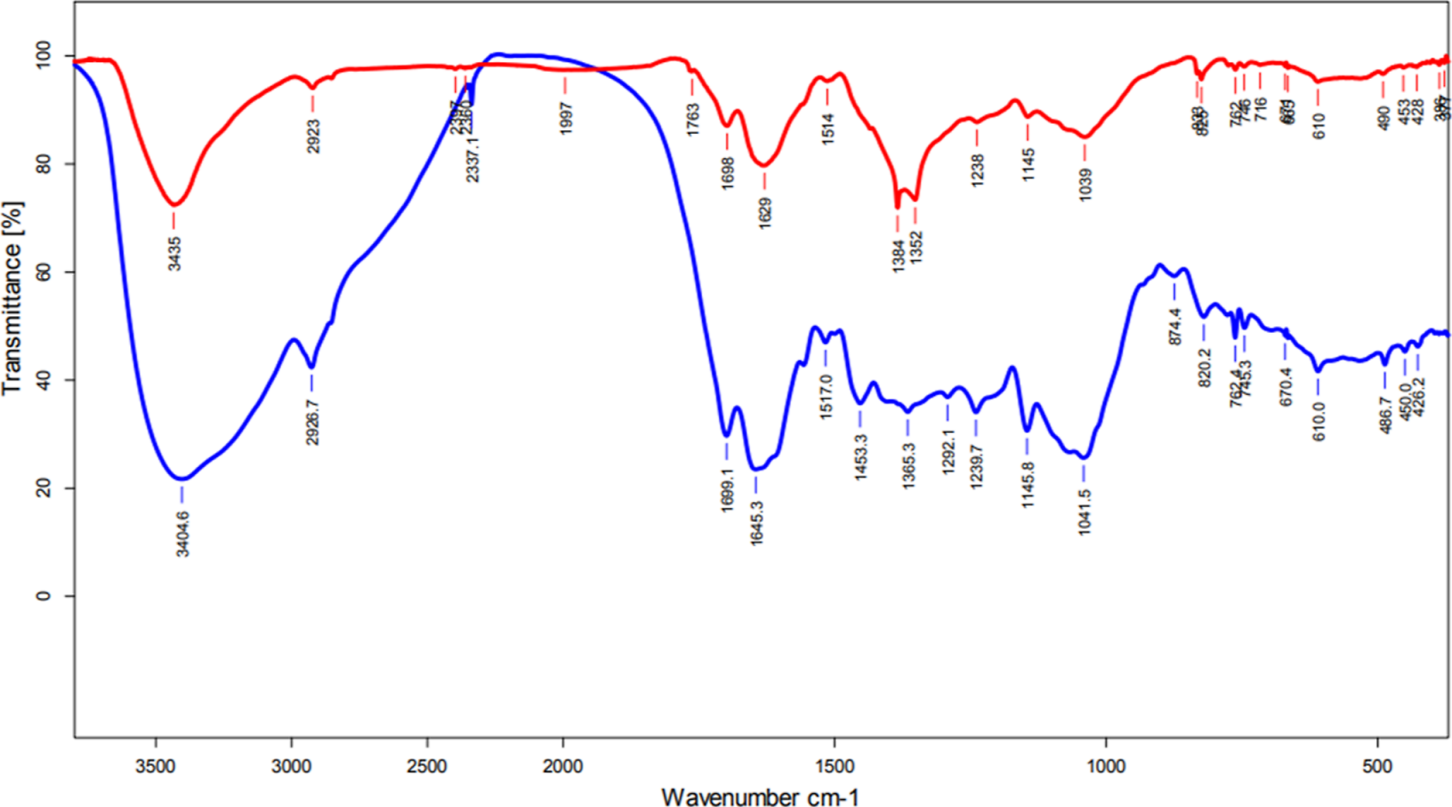
FTIR spectra for aqueous green tea extract (blue line) and aqueous green tea extract-mediated AgNPs (red line).

FTIR measurements were taken to further confirm these predictions. Interpretation of the FTIR analyses consisted of comparing the presence and location of bands corresponding to specific functional groups belonging to various bioactive compounds contained in the green tea extract with those observed in the sample measured after AgNP synthesis (Figure 8). This allows us to indicate those biomolecules that show reducing properties toward metal ions, thus leading to the synthesis of nanoparticles. At the same time, they also gain responsibility for ensuring the stability of nanoparticles by binding and interacting with their surface.

The FTIR spectrum for the aqueous green tea leaf extract was represented by a blue line (Figure 8). The shift at 3405 cm^–1^ probably came from the stretching vibration of the O–H bond in free OH groups found in the structure of catechins, phenols, and alcohols, but it can also be attributed to the stretching vibration of the N–H bond in amine. At 2927 cm^–1^, a band corresponding to the vibration of the C–H bond was observed. The strong marked band at 1645 cm^–1^ is assigned to the stretching vibration of the C=O or C–N bonds present in compounds like gallic acid, catechin, or theanine. The presence of a peak in the position of 1042 cm^–1^ informed us about the vibrations of the C–OH bonds belonging to alcohols or proteins. In the FTIR spectrum of green tea extract, bands at 820 and 610 cm^–1^ were also found, corresponding to out-of-plane vibrations attributed to aromatic C–H bonds and bending vibrations of the C=C bonds in alkenes, respectively.

The FTIR spectrum measured for the green tea-mediated AgNPs sample is marked with the red line (Figure 8). Detailed analysis confirmed the presence of bands corresponding to those found in the extract with slight shifts in the position of the peaks, which were located at 3435, 2923, 1040, 825, and 610 cm^–1^. Other representative bands were observed at 2360 cm^–1^ and attributed to a stretching vibration from a bond in the COOH group and also at 1629 cm^–1^, which corresponds to the vibration of bonds found in carboxylic acids. A particularly important peak is the one present at 1384 cm^–1^, which confirms the presence of bending vibrations assigned to phenolic C=O groups formed as a result of the simultaneous oxidation of hydroxyl groups and reduction of Ag^+^ ions to the Ag^0^ form. On the other hand, a small peak located at a wavelength of 386 cm^–1^ on the FTIR spectrum of the sample after nanoparticle synthesis is considered to identify the formation of silver metal. This is in line with previously recorded FTIR spectra for nanoparticles synthesized using tea extracts by other authors.^50,53,62,76^

Additionally, it should be underlined that the obtained FTIR results are in agreement with our earlier predictions and suggest that polyphenolic compounds determined by HPLC: EGCG and ECG and their interactions with Ag^+^ ions confirmed by FTIR analyses can play a double role: act as reducing agents in the synthesis of AgNPs and also stabilize them.

**Figure 9:**
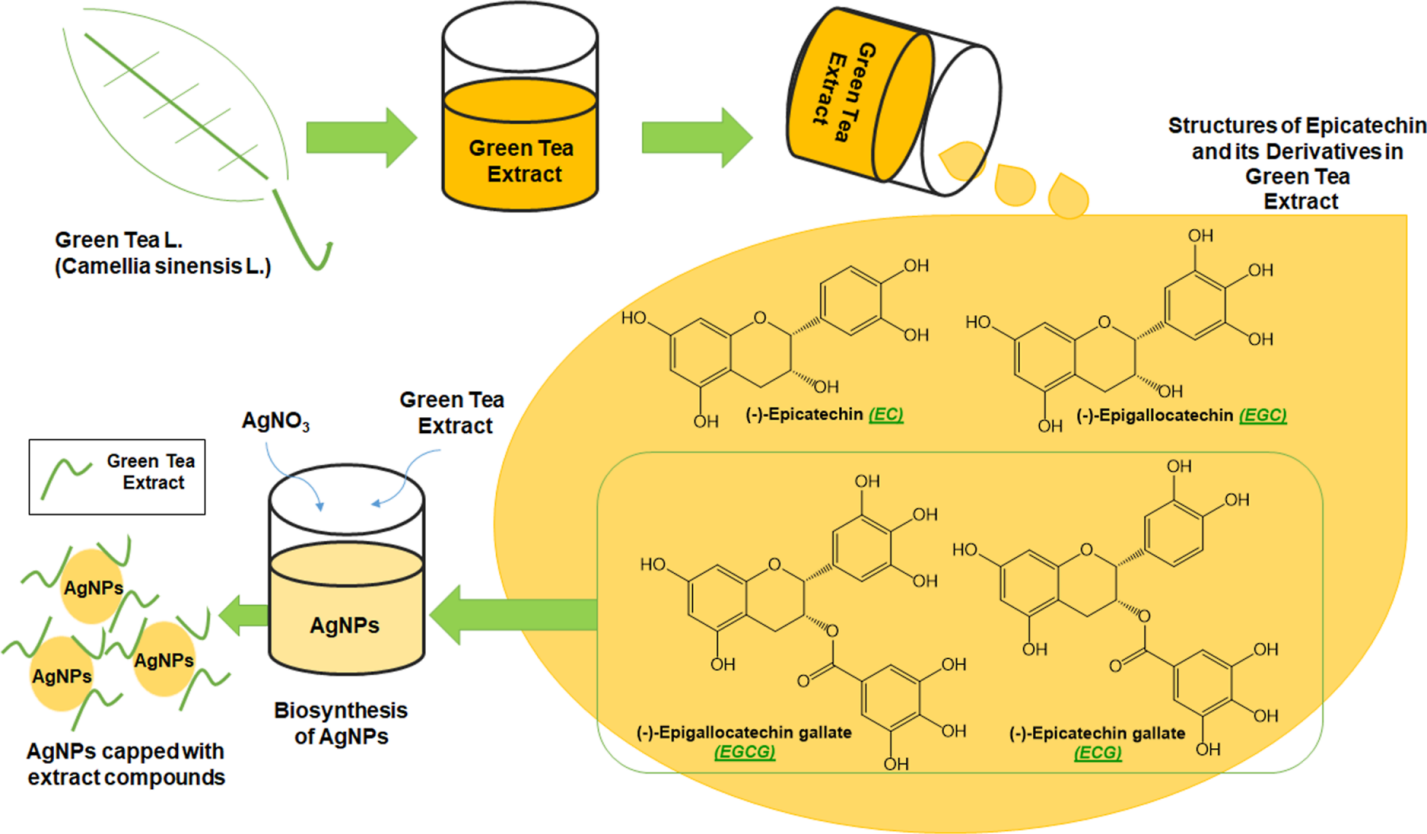
General scheme for the formation of AgNPs, including the chemical structures of epicatechin and its derivatives found in green tea extract.

The general scheme below (Figure 9) includes the chemical structures of all the biomolecules identified through the study that attend the biosynthesis process: epicatechin and its derivatives, especially EGCG and ECG, or determine the stability of AgNPs.

**Figure 10:**
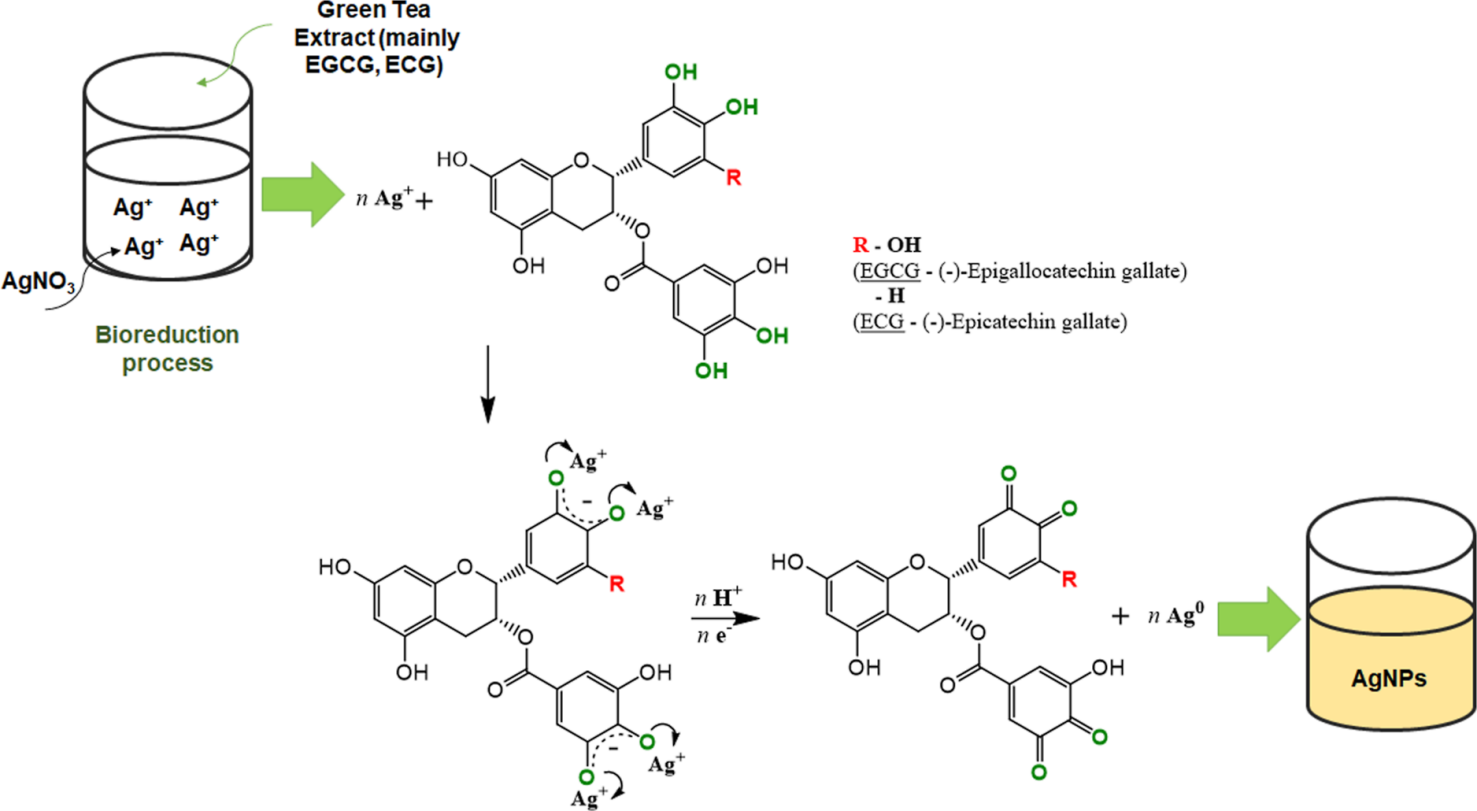
Probable course of bioreduction of the silver precursor (AgNO_3_) to AgNPs by two main components of green tea extract: EGCG and ECG.

A predicted scenario of changes in the functional groups of the two main components determined in the green tea leaf extract due to a specific mechanism of reaction with silver ions is proposed in Figure 10. In this way, it was possible to emphasize the distinctly high activity of plant polyphenols in the mechanism of nanoparticle formation, which is consistent with the previous literature examples.^74,94,95^

It can be seen that the mechanism presented above assumes the existence of two main stages, leading finally to the reduction of Ag^+^ ions to nanosilver. The presence of silver ions adjacent to polyphenols considered to be important reducing agents: EGCG and ECG leads to breaking the existing O–H bonds in hydroxyl groups attached to the aromatic ring belonging to the catechol fragment of biomolecules. As a consequence, an enol intermediate is formed, and then silver ions can be bound. Due to the low stability of this compound, the bond formed can be easily broken, which will occur with the simultaneous transfer of an electron to the metal ion, which is associated with its reduction to the Ag(0) form. This is evidenced by the presence of nanoparticles observed with TEM images. The reduction of metal ions also entails the formation of keto groups due to the oxidation of hydroxyl groups.

**Figure 11:**
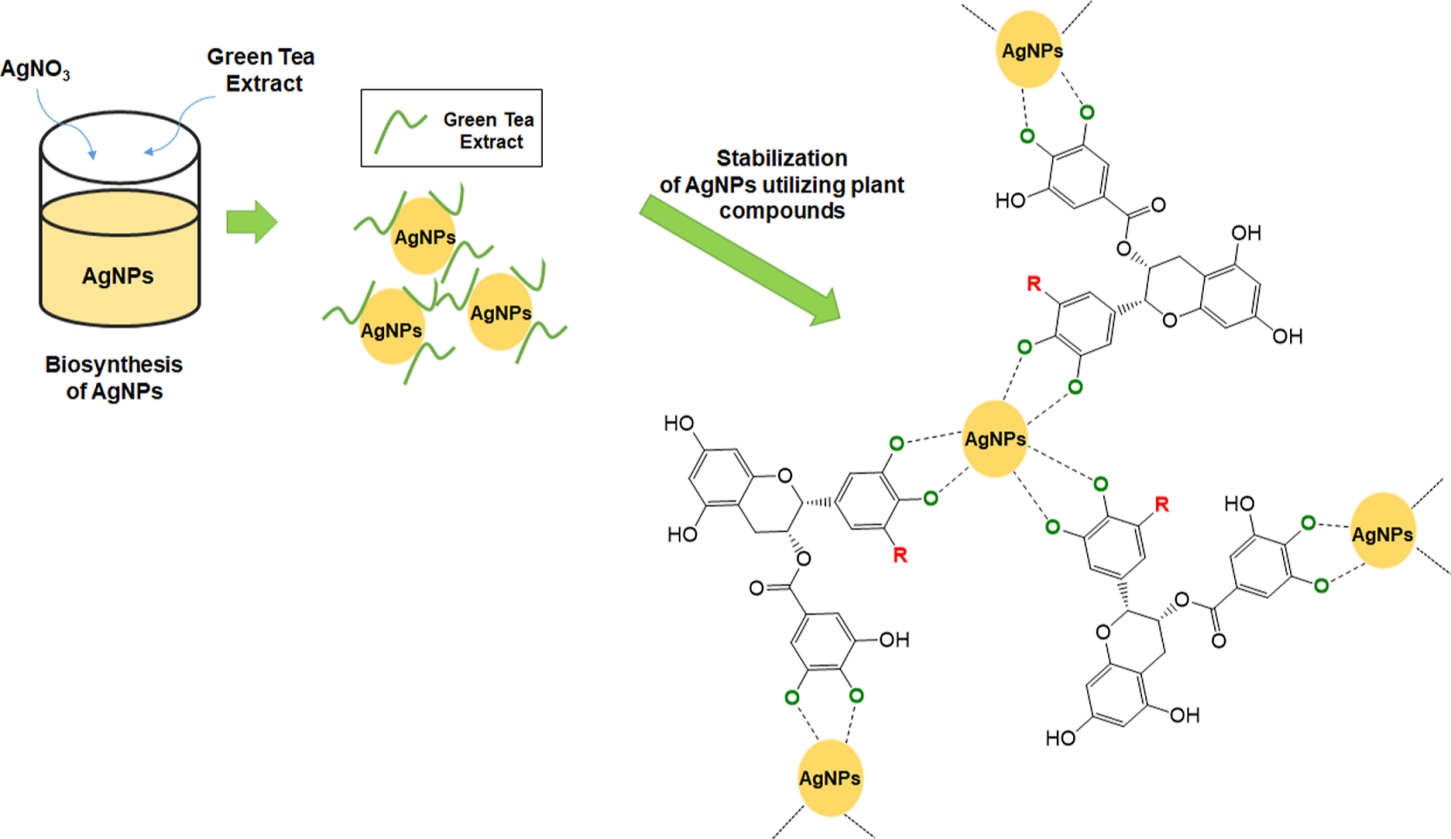
General scheme exemplifying the effect of stabilizing AgNPs by polyphenols found in green tea extract: EGCG and ECG.

As mentioned before, the biomolecules present in the extract also have the function of controlling the stability of nanoparticles through interactions occurring on their surface. It was confirmed using FTIR spectra. A predicted schematic image of such stabilization is shown in Figure 11.

The stabilization process is important from the point of view of avoiding the formation of aggregates as a result of covering the surface of nanoparticles with enol forms of biomolecules established in place of forms containing initially hydroxyl functional groups and, as a consequence, the appearance of suitable interactions between them and active sites on the surface of AgNPs.

## 4 Conclusions

In this paper, we focused on comparing the optimization of the extraction process of polyphenolic compounds from green tea leaves (*C. sinensis*) using two types of extractants: aqueous and hydroethanolic (ethanol/water ratio = 1:1). The optimization of the extraction based on the Box–Behnken statistical model’s matrix was successfully carried out in both cases using three factors as independent variables: pH, time, and temperature. The ranges for these levels were selected through single-factor experiments.

The total polyphenol content (TPC) was used as a surface response and determined in milligrams of gallic acid equivalent (GAE) per gram of dried biomass. TPC values obtained during extraction in the water/ethanol system were higher than those in water. As a result of optimization, two models with high experimental data fit to the model predictions were obtained. This allowed us to determine the optimal extraction conditions based on 3D response surface plots with different values for both extraction systems. The optimal conditions for water extraction were determined as follows: a temperature at 80 °C, a pH of 7.0, and a processing time of 30 min. The optimal parameters for the ethanol/water mixture extraction were the temperature at 80 °C, a pH of 5.5, and a duration time of 30 min. Both extracts were employed for synthesizing AgNPs; however, their physicochemical analysis revealed a tendency for partial nanoparticle aggregation in the hydroethanolic extract. It is important to note that this phenomenon does not occur when an aqueous extract is used. The differences in the bands observed through FTIR spectra of the extract and AgNP suspension samples allowed us to identify modifications occurring in the hydroxyl functional groups of polyphenols found in green tea leaf extract. Their final oxidation to ketone groups, being the result of the observed reduction of Ag^+^ ions to Ag(0) form, enabled us to propose a scheme for the mechanism of reduction and show the predicted course of nanoparticle stabilization. In conclusion, an ethanol/water mixture is a better solvent system for polyphenol extraction than water alone. However, for AgNP synthesis, it is preferable to use an aqueous extract, which, despite its lower concentration of polyphenols, does not lead to agglomeration. The aggregation of AgNPs can be attributed to the increased amount of organic macromolecules in the water–ethanol extract and changes in the reaction mixture’s solution parameters.

## 5 Future Perspectives

The main challenge for nanobiotechnologists and nanotechnologists is the development of a laboratory and, consequently, a pilot protocol for the synthesis of stable, durable, and nontoxic nanomaterials through biosynthesis procedures, including the extraction of plant components and then biosynthesis of NPs. Biological methods for the synthesis of NPs are now a key approach to replacing current chemical reduction methods, which are not environmentally or human health beneficial. Due to the fact that NPs, as the main building block of nanomaterials obtained with the assistance of plant material, often show brief stability in relation to shape and size. In addition, there is often a tendency to form aggregates during storage. Therefore, it would be appropriate to focus on finding nontoxic, affordable, and green stabilizing agents. The structure of the stabilizer should be designed based on the anticipated use of the nanomaterial and, preferably, where possible, use components that occur in nature as renewable resources. Here, interest should be turned toward ionic liquids, whose range of properties is wide. A major challenge is also posed by the absolutely unpredictable composition of biomolecules contained in plants, a large number of which have not yet been identified. Owing to the fact that the mechanism of reduction should be studied on each plant separately, with the goal of determining the biomolecules responsible for the reduction and stabilization of NPs. In order to maintain the low economic and time investment envisioned for a process, it is planned for implementation on an industrial production scale. It is important to direct further research toward optimizing processes based on statistical models. In our opinion, this trend is gaining popularity at the moment. It represents an opportunity to obtain the final reaction conditions at which performance is highest by dispensing with the need for a tedious series of experiments against each individual factor in favor of a few specific ones based on the most important parameters, as we mentioned in our article.

Therefore, the continuation of the research presented in this publication should be seen in attempts to increase the stability of nanoparticles by using suitable green stabilizers in the form of selected ionic liquids for both aqueous and ethanolic extracts, where the tendency to aggregation is evident. It is also possible to consider an optimization approach for the biosynthesis of nanoparticles in the presence of a stabilizer. Then the development of such a procedure from the point of view of application research will provide a starting point for research for antimicrobial, medical, and even catalytic applications. It is important to fulfill the conditions relating to the principles of Green Chemistry and sustainable development simultaneously.

## Supporting Information Available

Size distribution of particles of dried green tea leaves after grinding; absorbance values at λ = 750 nm for different concentrations of standard gallic acid solutions in water; calibration curve for different concentrations of gallic acid in water as an extraction solvent; absorbance values at λ = 750 nm for different concentrations of standard gallic acid solutions in ethanol; calibration curve for different concentrations of gallic acid in an ethanol/water mixture as an extraction solvent; Pareto graph of the standardized effect of independent variables and interaction between them at the aqueous extraction of Green Tea L; comparison of experimental and theoretical data for the aqueous extraction of polyphenols from Green Tea L; Pareto graph of the standardized effect of independent variables and interaction between them on TPC in hydroethanolic solvent extraction; comparison of experimental and theoretical data for hydroethanolic extraction of polyphenols from Green Tea L; predicted value of optimal parameter and desirable response as TPC value after extraction of Green Tea L. in water; predicted value of optimal parameter and desirable response as TPC value after extraction of Green Tea L. in ethanol/water mixture; and HPLC analysis results for aqueous and hydroalcoholic extracts (PDF)
