# Supplementary material for: Green Synthesis of Silver Nanoparticles: Optimizing Green Tea Leaf Extraction for Enhanced Physicochemical Properties
Source: ACS Omega. 2023 Aug 10;8(33):30532–49. doi: 10.1021/acsomega.3c03775 (PMC10448680; doi:10.1021/acsomega.3c03775)
Supplement: Supplementary file 1 — ao3c03775_si_001.pdf [file ao3c03775_si_001.pdf]

Supporting Information

Green Synthesis of Silver Nanoparticles: Optimizing Green Tea Leaf Extraction for Enhanced Physicochemical Properties

Anna Wirwis[a], Zygmunt Sadowski[a*]

[a]*Department of Process Engineering and Technology of Polymer and Carbon Materials*, Faculty of Chemistry, Wrocław University of Science and Technology, Wybrzeze Wyspianskiego 27, 50-370 Wrocław, Poland.

E-mails: anna.wirwis@pwr.edu.pl; zygmunt.sadowski@pwr.edu.pl;

*__Correspondence to__:

Anna Wirwis

E-mail address: anna.wirwis@pwr.edu.pl

Telephone: +48 71 320 29 75

Fig. S.1 Size distribution of particles of dried green tea leaves after milling in coffee grinder.

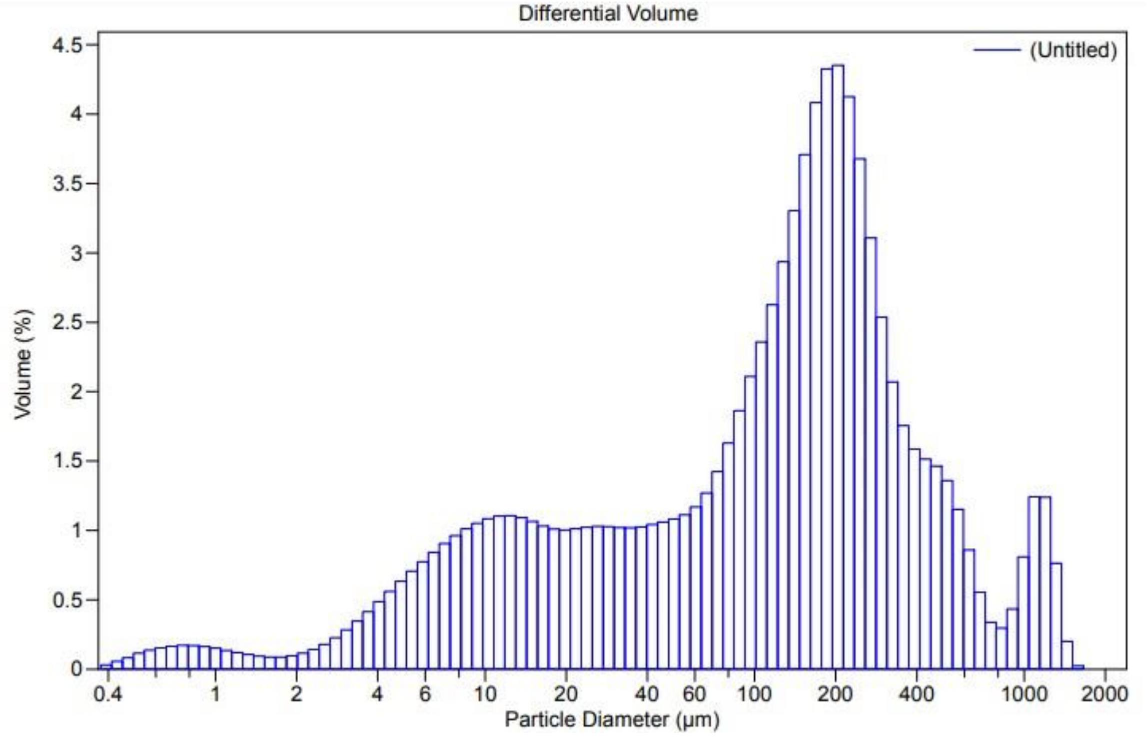

Table S.1 The absorbance values at λ=750 nm for different concentrations of standard gallic acid solutions in water.

Absorbance (A)	C[µg/ml]
0,029	10
0,054	20
0,088	30
0,114	40
0,151	50
0,168	60
0,240	80
0,297	100

Fig. S.2 Calibration curve for different concentration of gallic acid in water as an extraction solvent.

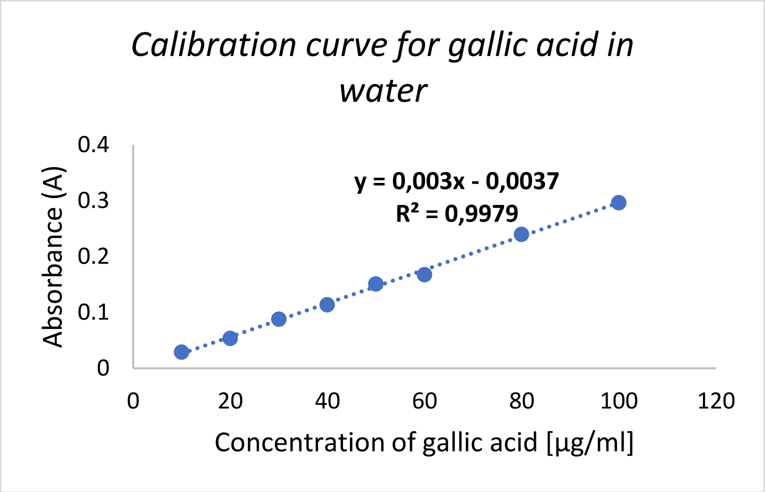

Table S.2 The absorbance values at λ=750 nm for different concentrations of standard gallic acid solutions in ethanol.

Absorbance (A)	C[µg/ml]
0,073	10
0,107	20
0,237	50
0,344	70
0,464	100

Fig. S.3 Calibration curve for different concentration of gallic acid in ethanol/water misture as a extraction solvent.

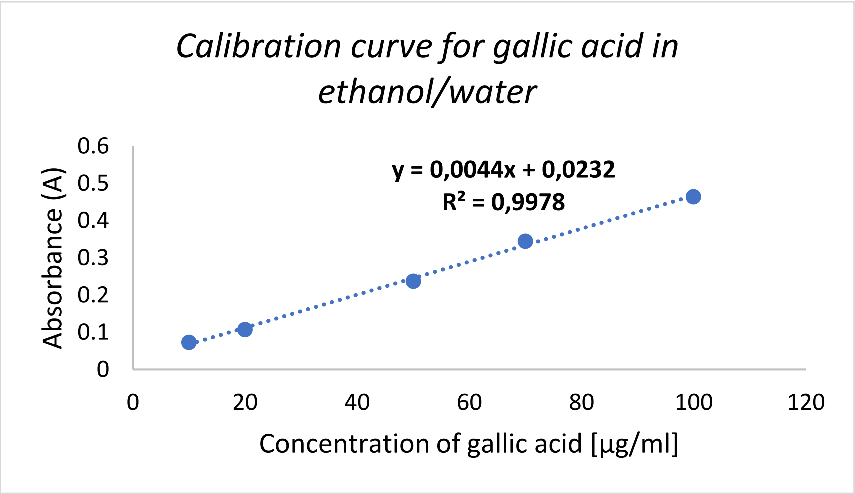

Fig. S.4 Pareto graph of the standardized effect of independent variables and interaction between them at the aqueous extraction of Green Tea L.

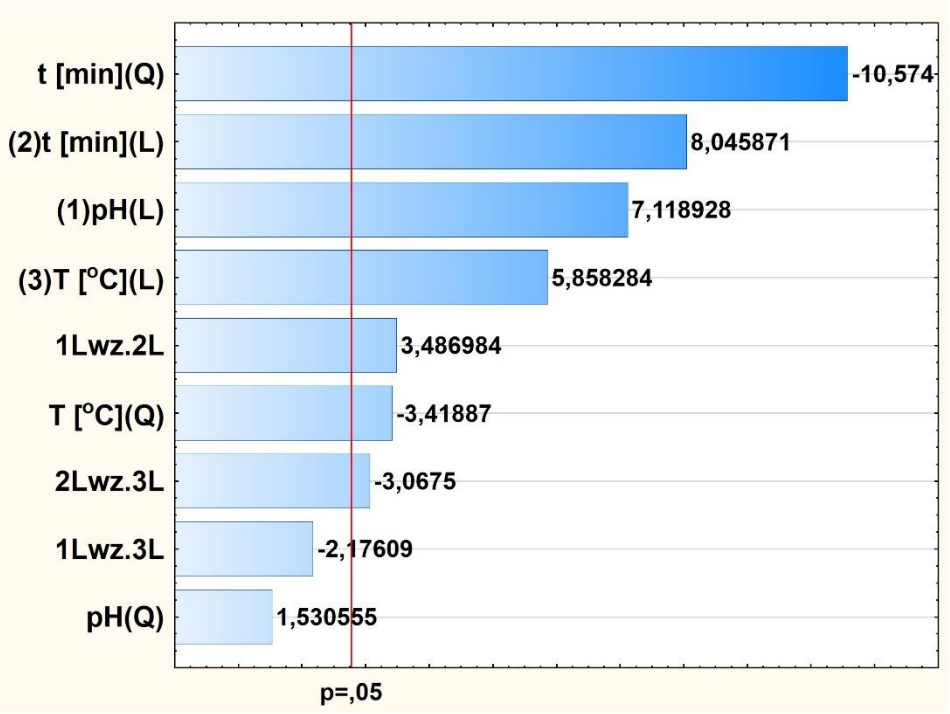

Fig. S.5 Comparison of experimental and theoretical data for aqueous extraction of polyphenols from Green Tea L.

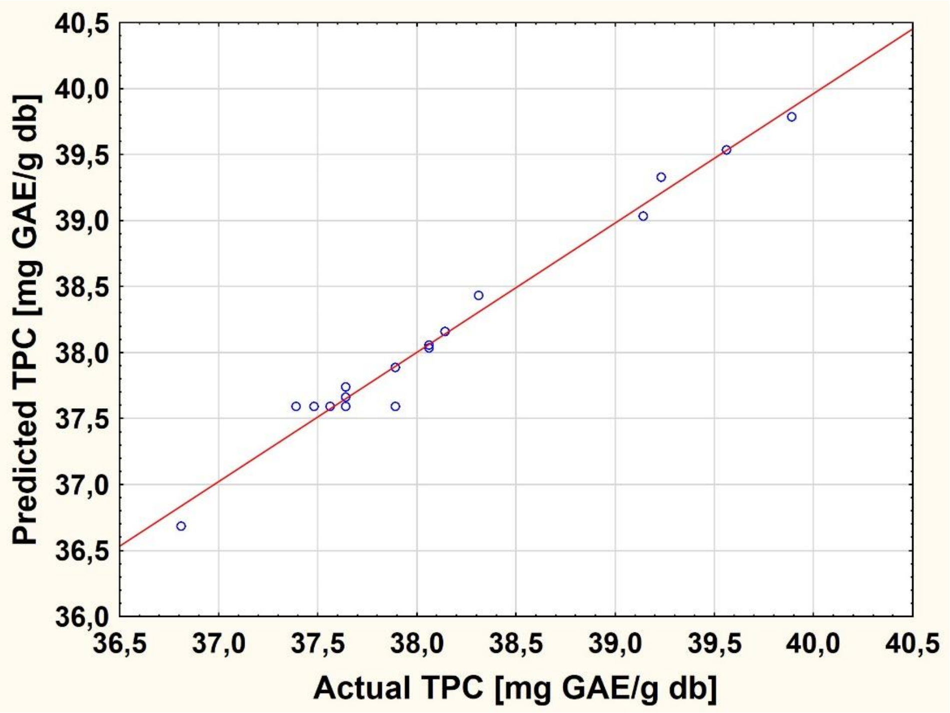

Fig. S.6 Pareto graph of the standardized effect of independent variables and interaction between them on TPC in hydroethanolic solvent extraction.

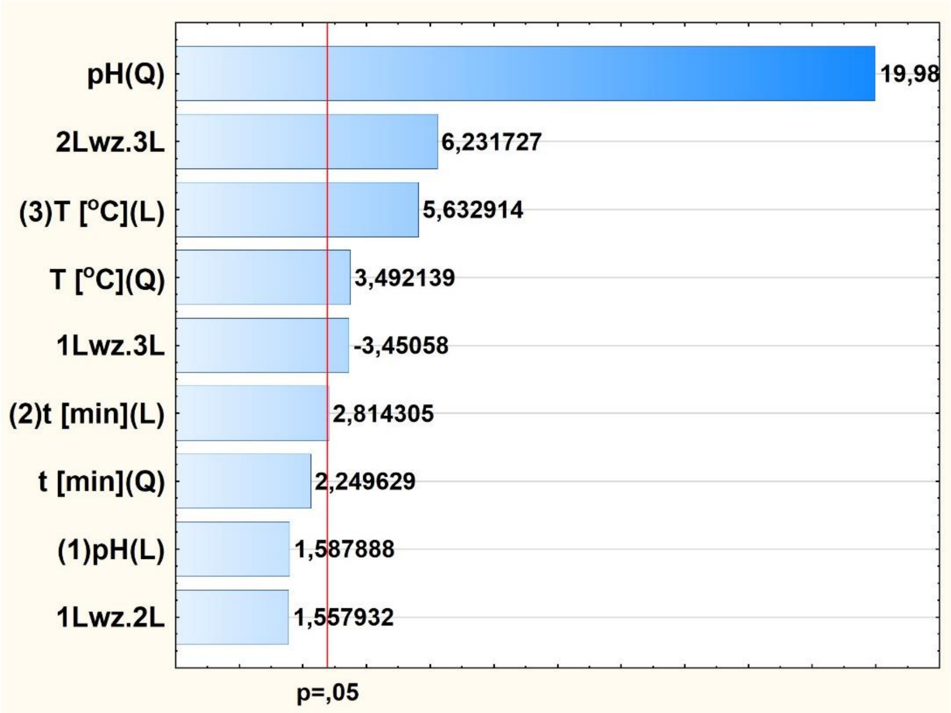

Fig. S.7 Comparison of experimental and theoretical data for hydroethanolic extraction of polyphenols from Green Tea L.

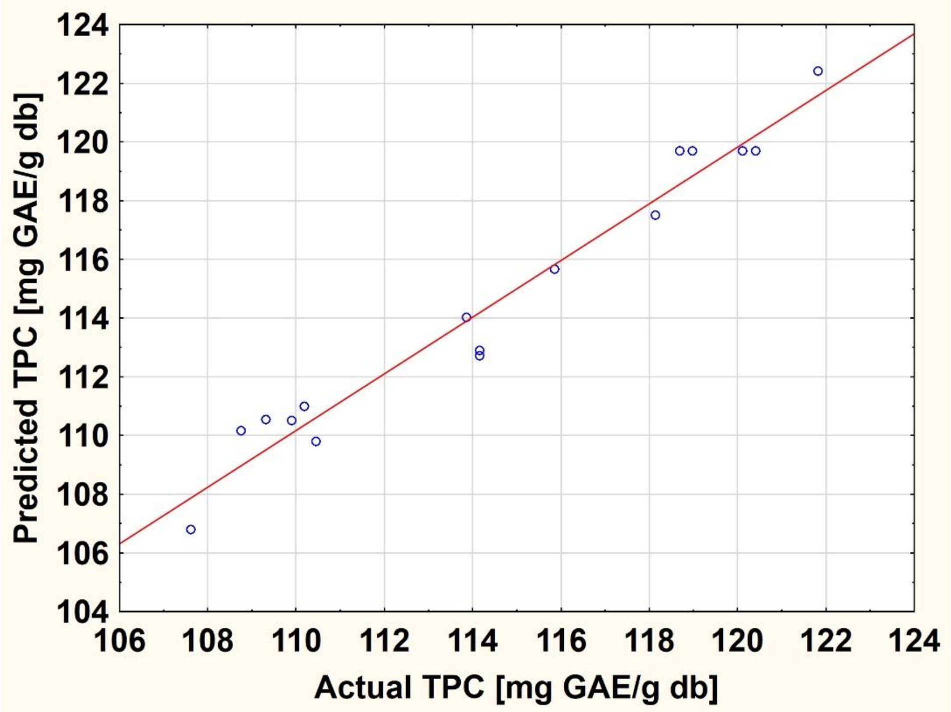

Fig. S.8 Predicted value of optimal parameter and desirable response as TPC value after extraction of Green Tea L. in water.

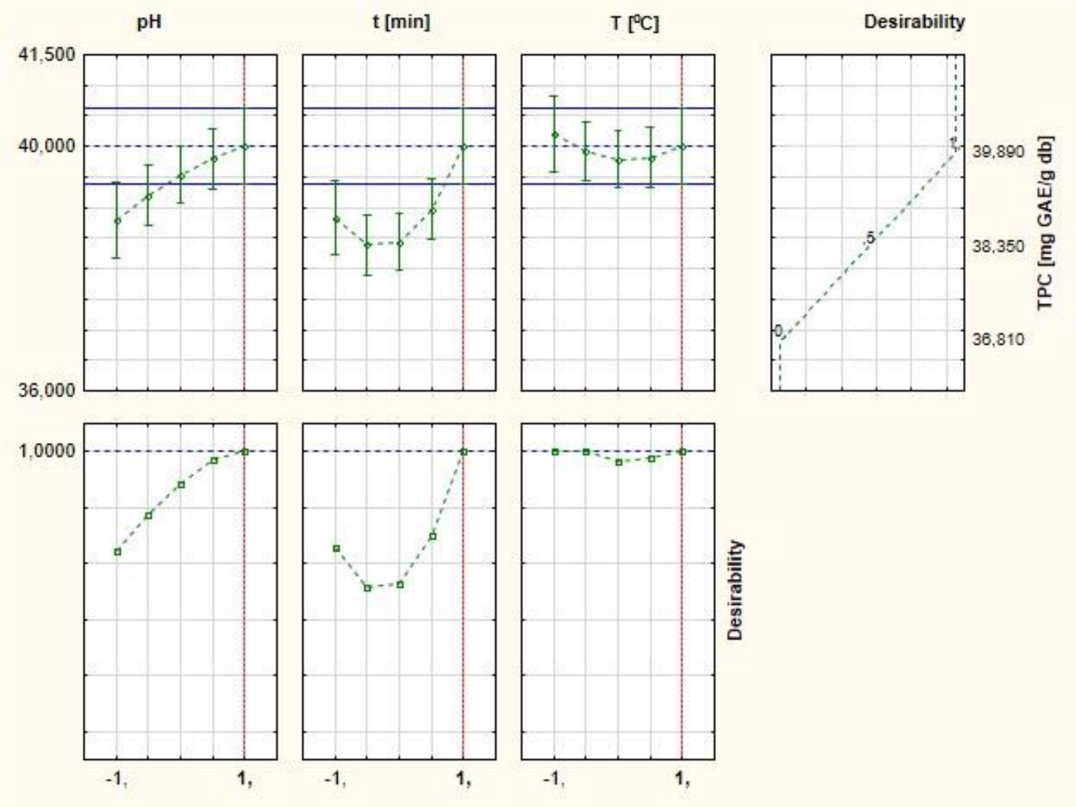

Fig. S.9 Predicted value of optimal parameter and desirable response as TPC value after extraction of Green Tea L. in ethanol/water mixture.

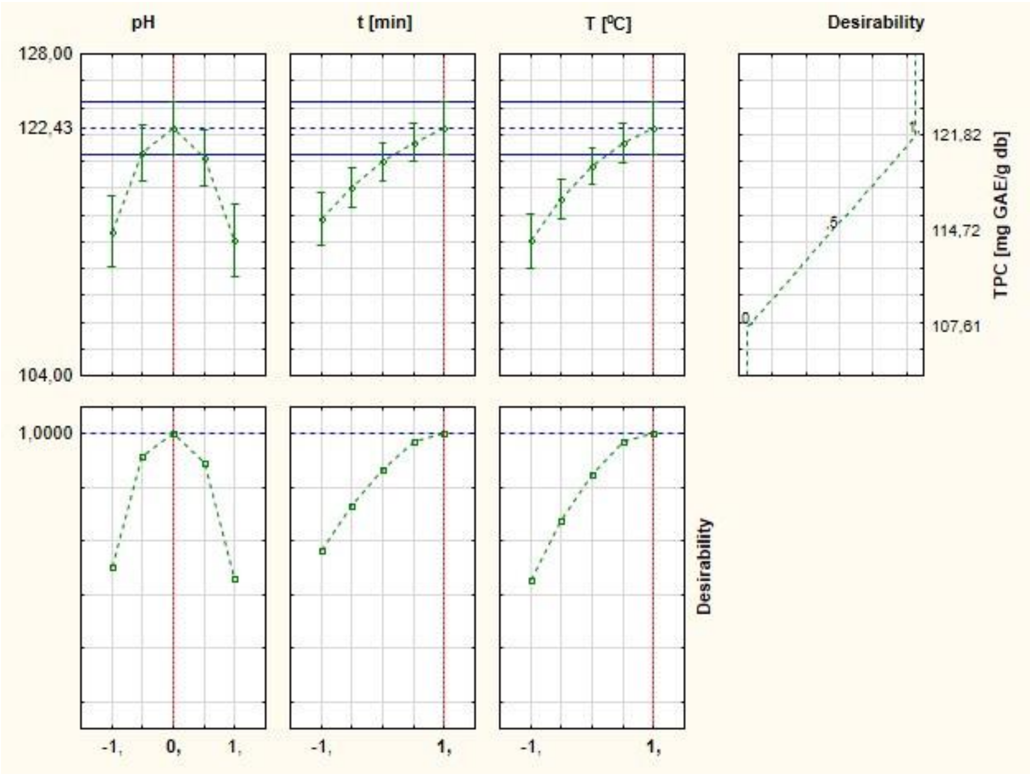

Table S.3 HPLC analysis results for aqueous and hydroalcoholic extracts

Extract components	Water	Water - ethanol
(-) Epigallacatechin (EGC)	2.38	0
Coffeine	52.24	37.52
(-) Epicatechin (EC)	4.08	2.19
(-) Epigallocatechin gallate (EGCG)	31.28	43.37
(-) Epicatechin-3-gallate (ECG)	10.02	20.04
